# Hypothalamus Participates in Peripheral Sympathetic Hyperactivity After Acute Brain Injury by Regulating *Trhr* M^6^A Modification

**DOI:** 10.1002/mco2.71036

**Published:** 2026-10-05

**Authors:** Maoxing Fei, Handong Wang, Chaochao Gao, Shiqiao Luo, Xiwen Huang, Yanling Han, Mengliang Zhou, Zhenxing Li

**Affiliations:** ^1^ Department of Neurosurgery Jinling Hospital Affiliated Hospital of Medical School Nanjing University Nanjing China; ^2^ Department of Neurosurgery Northern Jiangsu People's Hospital Affiliated to Yangzhou University Yangzhou China; ^3^ Department of Neurosurgery The Affiliated BenQ Hospital of Nanjing Medical University Nanjing China; ^4^ Department of Neurosurgery The Affiliated Jinling Hospital of Nanjing Medical University Nanjing China

**Keywords:** acute brain injury, fat mass and obesity‐associated gene, N^6^‐methyladenosine, sympathetic hyperactivity, thyrotropin‐releasing hormone receptor

## Abstract

Sympathetic hyperactivity following acute brain injury (ABI) impedes recovery, yet effective treatments are lacking. Recently, N^6^‐methyladenosine (m^6^A) modified by fat mass and obesity‐associated gene (FTO) has been demonstrated to play critical roles in multiple neurological diseases. Here, we found that sympathetic hyperactivity occurred in traumatic brain injury and intracerebral hemorrhage patients and mouse models in vivo. In mouse ABI models, the RNA demethylase FTO was upregulated in hypothalamic neurons. Through m^6^A methylation sequencing, we found that FTO mediated m^6^A demethylation of *Trhr* mRNA, enhancing its stability by inhibiting YTHDF2‐dependent decay. This increased neuronal TRHR expression and sensitivity to TRH, culminating in sympathetic hyperactivity. Conversely, restoring hypothalamic FTO or TRHR levels alleviated this condition. We further mapped and functionally validated a monosynaptic projection from lateral hypothalamic TRHR neurons to the rostral ventrolateral medulla (RVLM), demonstrating that modulating these neurons controls sympathetic tone. Collectively, our findings elucidate a novel FTO–Trhr axis in post‐ABI autonomic dysfunction and highlight a promising therapeutic target.

## Graphical abstract

In acute brain injury (TBI/ICH), sympathetic hyperactivity is driven by hypothalamic FTO upregulation. FTO demethylates *Trhr*, blocking m^6^A‐YTHDF2‐dependent degradation, which stabilizes *Trhr* mRNA and increases neuronal sensitivity to TRH. Restoring FTO or Trhr alleviates sympathetic hyperactivity, offering a potential target for managing autonomic dysfunction after ABI.

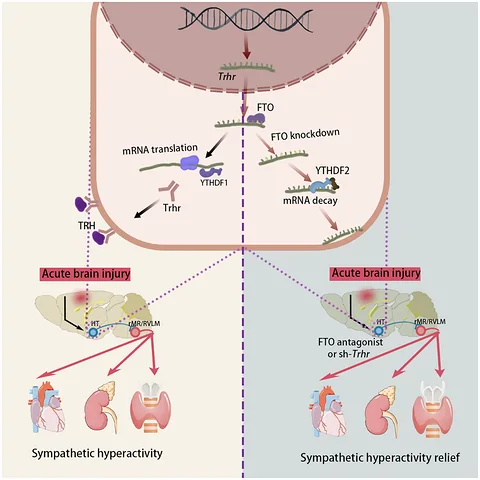

## 1 Introduction

The autonomic nervous system maintains normal communication between the brain and peripheral organs, such as the heart, spleen, and intestines [1, 2, 3]. However, autonomic dysfunction frequently occurs after acute brain injury (ABI), often presenting as sympathetic hyperactivity. This hyperactivity not only exacerbates the primary brain injury but also leads to systemic complications, including cardiovascular and metabolic dysfunction [4]. Its most pronounced form is known as paroxysmal sympathetic hyperactivity (PSH), characterized by paroxysmal tachycardia, hypertension, tachypnea, hyperthermia, and decerebrate posturing [5]. PSH is a recognized acute complication in neurointensive care unit (NICU) patients and is associated with poor outcomes [6]. Clinical studies report that over 80% of PSH cases are associated with ABI, primarily traumatic brain injury (TBI), and intracerebral hemorrhage (ICH) [5]. Although empirical therapy for PSH relies on symptomatic management, no effective treatment currently exists to prevent its occurrence in the early stages following ABI. Therefore, elucidating the pathogenesis of sympathetic hyperactivity after ABI is crucial for developing effective treatment strategies.

The autonomic nervous system is an elaborate network that modulates whole‐body functions. The hypothalamus, a key regulatory center within this system, maintains essential homeostasis by regulating systemic hormones, metabolism, and behavior [7]. Previous studies have shown that physiological challenges (e.g., cold stress) or pathological conditions (e.g., hypothalamic inflammation) can induce metabolic and behavioral abnormalities via the hypothalamus [8, 9]. The hypothalamic–medullary circuit serves as a critical pathway for neural signaling, regulating sympathetic and parasympathetic outflow to influence heart rate and blood pressure [7]. Although the role of central nuclei in regulating peripheral organ function has gained increasing recognition, the underlying molecular mechanisms remain poorly understood. Given that increased heart rate and blood pressure instability are hallmarks of sympathetic hyperactivity [5], it is crucial to investigate how the hypothalamic–medullary axis is altered at the molecular level after ABI and its potential role in driving sympathetic hyperactivity.

N^6^‐methyladenosine (m^6^A), a pivotal post‐transcriptional epigenetic modification, is implicated in various central nervous system diseases. Notably, m^6^A‐related proteins are highly expressed in the brain [10], suggesting a critical role for this modification in neurobiology. Previous studies have linked dysregulation of neuronal m^6^A modification to depression, anxiety [11, 12], and thalamic pain following ICH [13]. However, it remains unknown whether hypothalamic neurons undergo m^6^A remodeling after ABI, and if so, whether such changes contribute to the development of sympathetic hyperactivity.

This study aims to elucidate the molecular mechanisms within hypothalamic neurons that underlie sympathetic hyperactivity after ABI. We focused on the role of RNA m^6^A modification, investigating how the demethylase FTO regulates thyrotropin‐releasing hormone receptor (Trhr) expression. Furthermore, we explored the impact of the FTO–Trhr pathway on hypothalamic–medullary circuitry to clarify its overall role in driving sympathetic hyperactivity.

## 2 Results

### 2.1 Sympathetic Hyperactivity in Patients With ABI

To assess autonomic function following ABI, we analyzed electrocardiogram (ECG) recordings from patients with TBI or ICH, comparing them with recordings from healthy participants (Figure 1A). No significant differences were found in baseline characteristics such as age or gender between the groups (Table S1). Heart rate variability (HRV) analysis revealed a significant increase in sympathetic tone and a concomitant decrease in parasympathetic tone in both ICH and TBI patients compared to controls (sympathetic tone index: ICH group, TBI group vs. control group, 10.08 ± 1.083, 11.08 ± 1.069 vs. 5.00 ± 0.5638, *p^ICH vs. Ctrl^* = 0.0005, *p^TBI vs. Ctrl^* < 0.0001; parasympathetic tone index: ICH group, TBI group vs. control group, −3.065 ± 0.1827, −3.233 ± 0.1790 vs. −2.186 ± 0.1775, *p^ICH vs. Ctrl^* = 0.0017, *p^TBI vs. Ctrl^* = 0.0002) (Figure 1B,C). Meanwhile, the stress index was also elevated in both patient groups (Figure 1D). Analysis of hematological parameters showed no significant differences in serum sodium levels among the groups (Figure 1E). However, blood glucose and pro‐BNP levels were significantly elevated in ICH and TBI patients compared to the control group (Figure 1F,G), consistent with a state of sympathetic hyperactivity that may contribute to cardiac and other end‐organ injury. Collectively, these findings from our clinical cohort indicate that ABI is associated with a significant risk of autonomic dysregulation, though more patient data are warranted to delineate a thorough picture of autonomic disorder under the condition of ABI.

**FIGURE 1:**
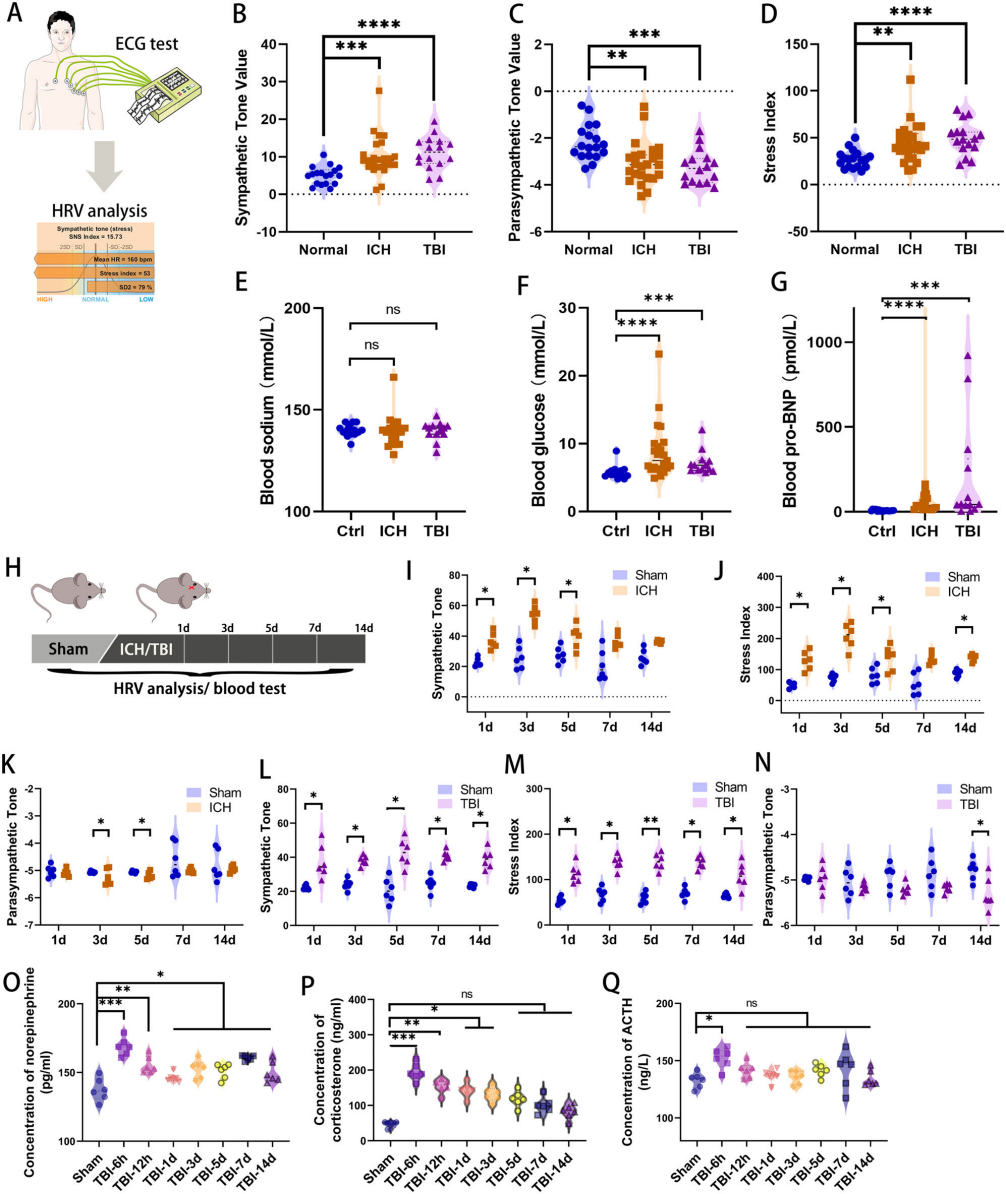
Sympathetic hyperactivity happens in ABI patients and ABI model in vivo. (A) Illustration of electrocardiogram data obtained from patients and normal participants and HRV analysis. (B–D) Sympathetic tone value (B), parasympathetic tone value (C), and stress index (D) were obtained by analyzing HRV. (E–G) The concentrations of blood sodium, blood glucose, and blood pro‐brain natriuretic peptide (pro‐BNP) in ABI patients and normal participants were collected and analyzed. (H) Illustration of model experiments in vivo. (I–N) Sympathetic tone value, stress index, and parasympathetic tone value of ICH (I–K) and TBI (L–N) models in vivo were obtained by analyzing HRV. *n* = 6. (O–Q) The concentrations of plasma norepinephrine, corticosterone, and adrenocorticotropic hormone (ACTH) from the sham group and the TBI groups were detected by ELISA. *n* = 6. ABI, acute brain injury. ICH, intracerebral hemorrhage. TBI, traumatic brain injury. Data are presented as mean ± SEM and were analyzed by Student's *t*‐test. ns represents no significance. ^*^ represents *p* < 0.05, ^**^ represents *p* < 0.01, ^***^ represents *p* < 0.001, and ^****^ represents *p* < 0.0001.

### 2.2 Sympathetic Hyperactivity in ABI Animal Models

To evaluate whether sympathetic hyperactivity also occurs in experimental models, we recorded and analyzed ECG data from mice with TBI or ICH (Figure 1H). HRV analysis using Kubios software revealed patterns consistent with those observed in patients: both TBI and ICH mice showed elevated sympathetic tone, reduced parasympathetic tone, and an increased stress index compared with sham‐operated controls (Figure 1I–N), although the changes in parasympathetic tone were less pronounced in the animal model than in the clinical data. Notably, these signs of sympathetic hyperactivity emerged early after ABI and persisted for at least 2 weeks (Figure 1I–N). Consistent with clinical findings, animal studies have shown that sympathetic hyperactivity is a common manifestation after ABI.

Activation of the hypothalamic–pituitary–adrenal (HPA) axis represents another hallmark of sympathetic hyperactivity. To assess adrenal activation, we examined the expression of the early response gene Per1, a known marker of adrenal cellular activity [14, 15]. Immunoblotting and immunofluorescence analyses showed that Per1 expression was significantly upregulated in the adrenal glands of TBI mice relative to sham controls (Figure S1A–D). Consistent with this, plasma norepinephrine (NE) and corticosterone levels were elevated in TBI mice and remained high throughout the subacute phase (Figure 1O,P). In contrast, plasma adrenocorticotropic hormone (ACTH) was only transiently increased at 6 h post‐TBI (Figure 1Q). Based on these differential temporal patterns, we hypothesize that the sustained activation of the adrenal gland following TBI may be mediated not only by humoral factors but also through direct neural innervation.

### 2.3 Altered mRNA m^6^A Modification in the Hypothalamus Following ABI In Vivo and In Vitro

The hypothalamus serves as a central regulator of systemic autonomic rhythms, largely mediated through its direct neural connections with the medulla oblongata  [7]. Using fluorescence staining of c‐FOS, we observed a marked increase in the number of c‐FOS^+^ neurons in the lateral hypothalamus area (LHA) after TBI, indicating neuronal activation in this region (Figure 2A,B). We further assessed neuronal activation in the medulla oblongata and found increased c‐FOS^+^ neurons in both the rostral medullary raphe (rMR) and rostral ventrolateral medulla (RVLM) following ABI (Figure S2A,B). Together with prior evidence, these results suggest that activation of the hypothalamic–ventrolateral medullary axis may underlie sympathetic hyperactivity after ABI.

**FIGURE 2:**
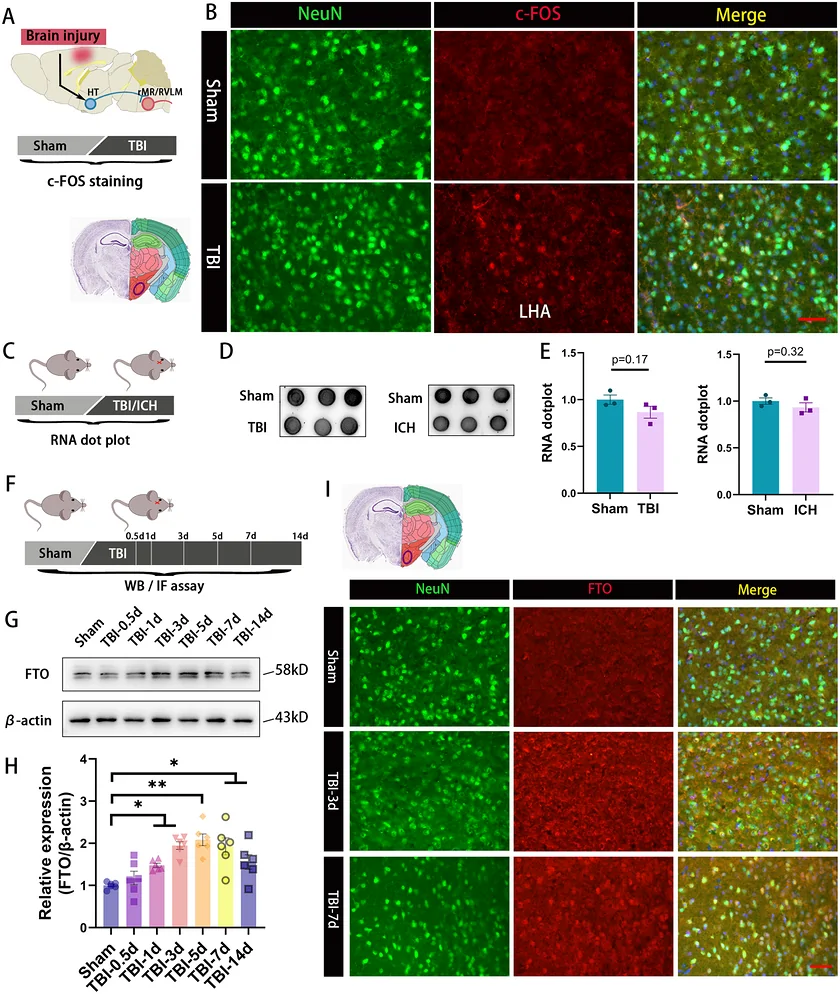
mRNA m^6^A modification changes happened in the hypothalamus after ABI in vivo. (A) Illustration of experimental process in vivo. (B) The hypothalamic region (lateral hypothalamus area, LHA) of the sham group and TBI group was stained with c‐FOS. Compared with the sham group, more c‐FOS‐positive neurons were observed in the TBI group. (C) Illustration of experimental process in vivo. (D and E) RNA dot blot was conducted using hypothalamic RNA extracts from the sham group, the TBI group, and the ICH group. m^6^A levels were slightly decreased in the TBI and ICH groups, though no significance was observed. *n* = 3. (F) Illustration of experimental process in vivo. (G and H) Image and analysis of the sham group and the TBI groups of different time points showed that protein expression of FTO increased in the TBI groups compared with the sham group. (I) Representative images of FTO immunofluorescence in the hypothalamic region of the sham group, the TBI‐3d group, and the TBI‐7d group. Compared with the sham group, more FTO‐positive neurons were observed in the TBI groups. *n* = 6. Data are presented as mean ± SEM and were analyzed by Student's *t*‐test. ns represents no significance. ^*^ represents *p* < 0.05 and ^**^ represents *p* < 0.01. Scale bars: 25 µm.

Given the established role of m^6^A as a prevalent mRNA epigenetic modification, we asked whether it participates in regulating hypothalamic transcripts in ABI. We first measured m^6^A levels in the mouse hypothalamus in TBI and ICH models (Figure 2C). RNA dot blot assays using an m^6^A‐specific antibody revealed a decreasing trend in hypothalamic m^6^A levels 3 days after TBI or ICH, though the change did not reach statistical significance (Figure 2D,E). This trend was consistent with elevated expression of the demethylase FTO in the TBI group, as shown by immunoblotting (Figure 2F–H). Immunofluorescence further confirmed increased FTO‐positive neurons in the hypothalamus at 3 and 7 days post‐TBI (Figure 2I). Interestingly, levels of the methyltransferases Mettl3 and WTAP were also elevated (Figure S3A–C). Collectively, these data indicate dynamic remodeling of m^6^A modification in the hypothalamus after ABI.

To corroborate these findings in a controlled setting, we assessed m^6^A levels and related proteins in primary neurons treated with oxyhemoglobin (Hb) to mimic hemorrhagic injury in vitro (Figure 3A). RNA dot blot showed a mild but consistent decrease in m^6^A levels upon Hb treatment (Figure 3B,C), paralleled by increased FTO protein expression detected by immunoblot assays (Figure 3D–F). Given reports linking FTO to neuronal responses under external stimuli [16, 17], we wonder whether FTO participates in neuronal activation. To test this, we knocked down FTO in primary neurons and evaluated activation markers (Figure 3G). Knockdown efficiency was confirmed by immunoblotting (Figure 3H,I). Hb treatment increased levels of c‐FOS and p‐ERK, but this effect was attenuated in FTO‐knockdown neurons (Figure 3J–L), suggesting that FTO contributes to neuronal activation. Meanwhile, Sholl analysis revealed that FTO knockdown in primary neurons did not affect neuronal morphology (Figure S3D). To visually monitor neuronal activity, we transduced neurons with a lentivirus encoding the fast calcium sensor jGCaMP7f (Figure 3M–O). At baseline, calcium signals did not differ between sh‐Ctrl and sh‐FTO groups (Figure 3M). However, upon stimulation with 20 µM Ca^2+^, sh‐FTO neurons exhibited a significantly lower calcium response than controls (Figure 3N,O), further supporting a role for FTO in promoting neuronal excitability.

**FIGURE 3:**
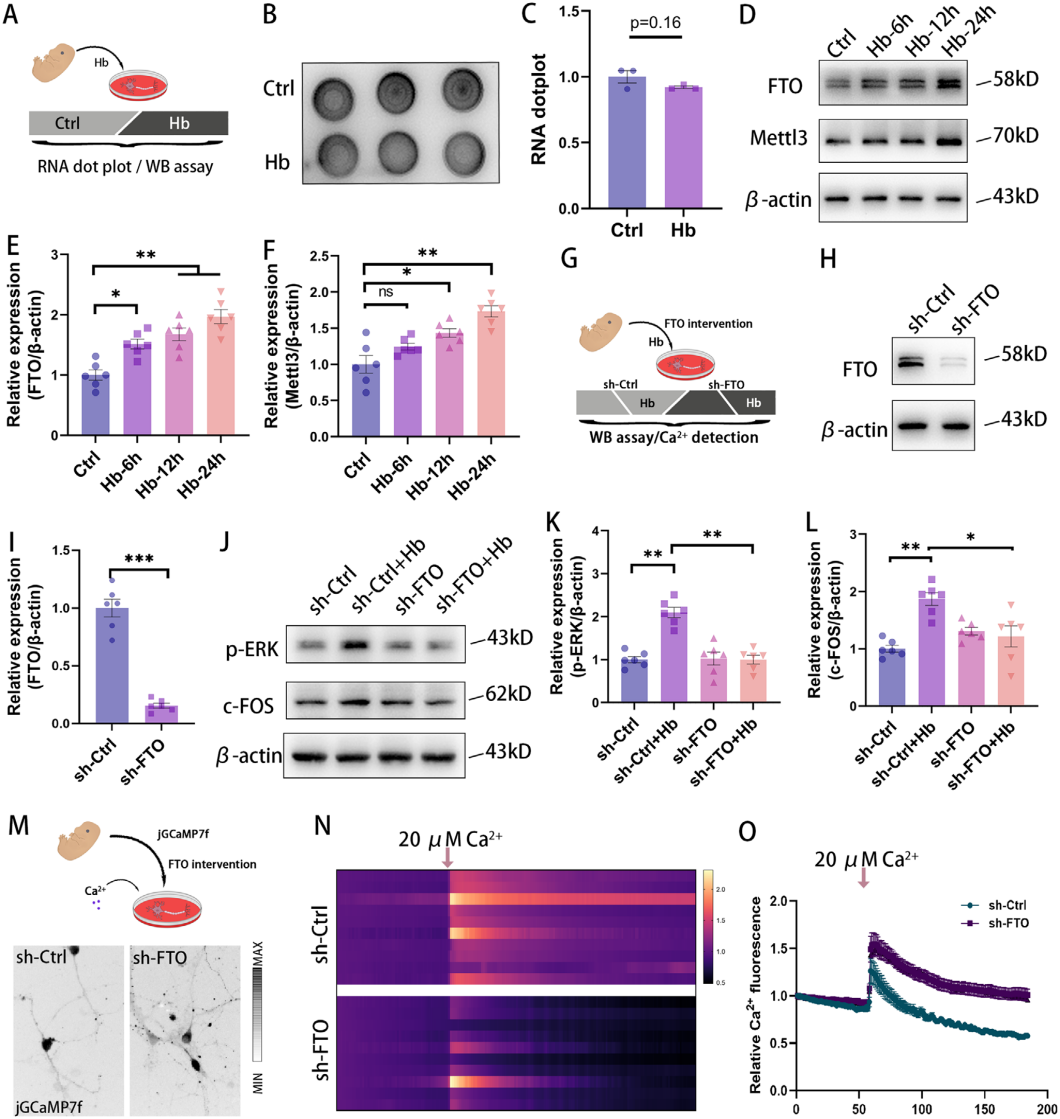
mRNA m^6^A modification changes happened in the primary neuron after oxyhemoglobin insult in vitro. (A) Illustration of experimental process in vitro. (B and C) Primary neurons were treated with oxyhemoglobin (Hb) and RNA dot blot assay showed that neurons experienced a subtly decreased m^6^A levels after the addition of Hb. (D–F) FTO and Mettl3 protein expression of the control group and Hb‐treated groups were detected by immunoblot, showing elevation of FTO and Mettl3 expression in the Hb‐treated groups. (G) Illustration of experimental process in vitro. (H and I) The knockdown efficiency of FTO was confirmed by immunoblot. (J–L) Representative pictures and analysis of immunoblot showed that neuron‐activation‐related proteins c‐FOS and p‐ERK were increased in the sh‐Ctrl+Hb group compared to the sh‐Ctrl group, while decreased in the sh‐FTO+Hb group compared to the sh‐Ctrl+Hb group. (M–O) Lentivirus encoding fast responsive Ca^2+^ fluorescent probe jGCaMP7f was developed to detect Ca^2+^ activity in neurons to reflect neural activity. Baseline Ca^2+^ picture showed that no obvious change was observed in the sh‐Ctrl group and the sh‐FTO group (M). Dynamic Ca^2+^ detection revealed neural activation among the sh‐Ctrl group and the sh‐FTO group (N and O). *n* = 6. Data are presented as mean ± SEM and were analyzed by Student's *t*‐test. ns represents no significance. ^*^ represents *p* < 0.05, ^**^ represents *p* < 0.01, and ^***^ represents *p* < 0.001.

### 2.4 mRNA Regulation by m^6^A Following Oxyhemoglobin Treatment

To delineate the molecular mechanism by which mRNA m^6^A modification regulates sympathetic hyperactivity after ABI, we performed methylated RNA immunoprecipitation sequencing (MeRIP‐seq) in primary neurons transduced with sh‐Ctrl or sh‐FTO lentivirus and treated with or without Hb (Figure S4A–D). The MeRIP‐seq results confirmed a modest decrease in overall m^6^A peaks in the Hb‐treated group compared to controls (Figure S4E). We identified 449 genes with significantly downregulated m^6^A peaks (fold change > 2) in the sh‐Ctrl+Hb group relative to the sh‐Ctrl group, among which 30 were differentially expressed at the transcript level (Figure S5A,B). Gene Ontology (GO) and Kyoto Encyclopedia of Genes and Genomes (KEGG) enrichment analyses indicated that these hypomethylated transcripts were functionally associated with damage response, neuronal repair, and thyroid hormone‐mediated signaling pathways (Figure S5A,B).

To further assess the effect of FTO knockdown on neuronal m^6^A modification under Hb treatment, we identified overlapping genes that exhibited both reduced m^6^A peaks in the sh‐Ctrl+Hb group (vs. sh‐Ctrl) and increased m^6^A peaks in the sh‐FTO+Hb group (vs. sh‐Ctrl+Hb) (Figure S5C). From this intersection, we selected 40 candidate genes—including *Slc36a4, Cccr2*, and *Trhr—*for further analysis. GO and KEGG analyses suggested that these genes are primarily involved in transmembrane transport and signal transduction (Figure S5C).

### 2.5 TRHR Is Identified as a Potential Mediator of Sympathetic Hyperactivity After ABI

Among the candidate genes (*Slc36a4, Cccr2*, *Trhr*…) identified by MeRIP‐seq, we focused on *Trhr*, which encodes the thyrotropin‐releasing hormone receptor (TRHR). We found that m^6^A levels of *Trhr* mRNA decreased upon Hb treatment, but increased upon FTO knockdown, suggesting that *Trhr* m^6^A modification is regulated by FTO after acute neuronal injury (Figure 4A,B). Previous studies have linked TRHR to autonomic functions such as stress response, heart rate, and blood pressure regulation [18, 19]. RT‑qPCR confirmed that *Trhr* transcript levels increased in the Hb‑treated group and decreased when FTO was knocked down (Figure 4C). Therefore, we speculate that FTO‑mediated demethylation of *Trhr* may contribute to sympathetic hyperactivity after ABI.

**FIGURE 4:**
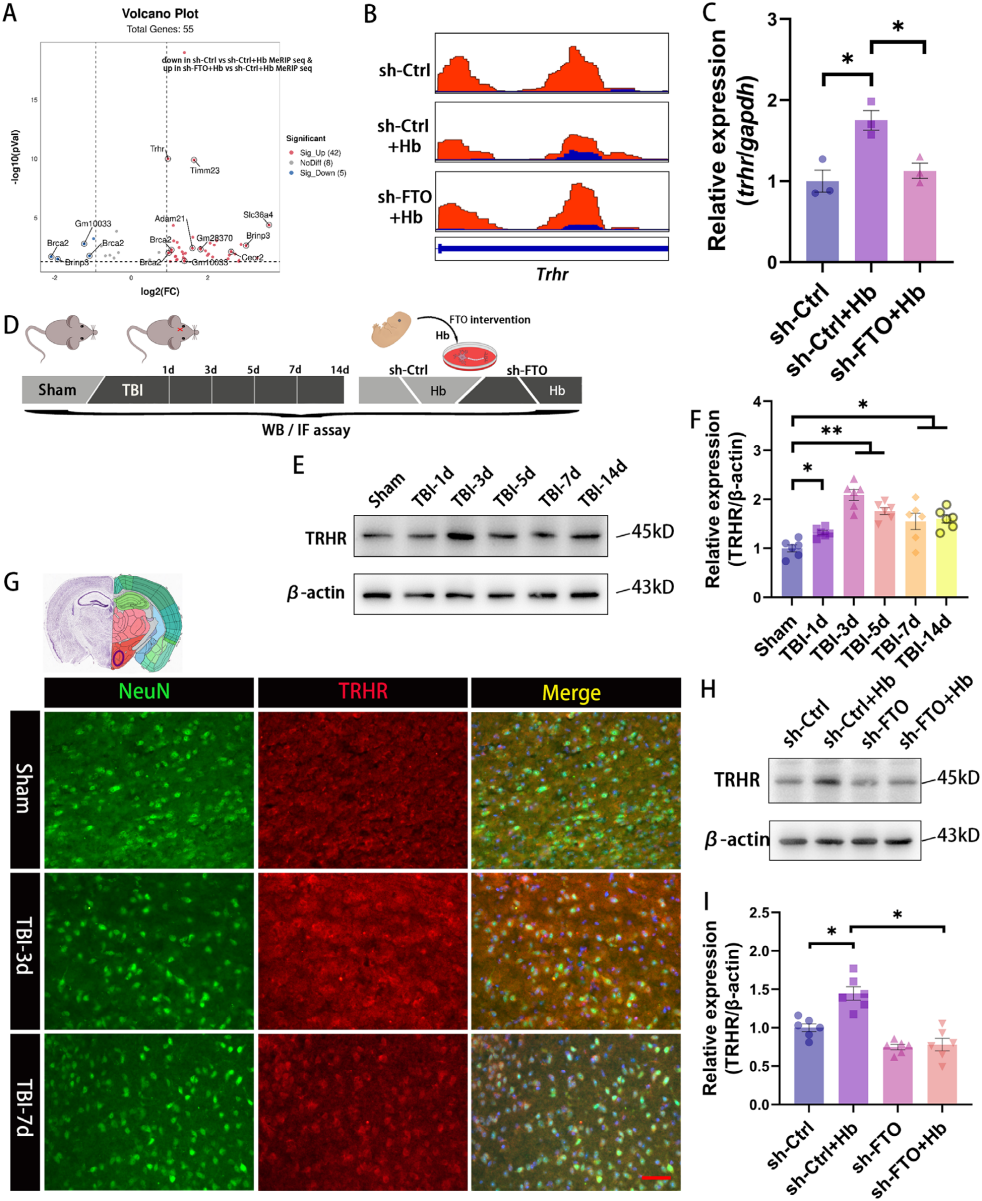
*Trhr* m^6^A modification is mediated by FTO. (A) Volcano plot exhibited genes in which m^6^A levels were downregulated in the Hb‐treated group compared to the control group and elevated in the sh‐FTO+Hb group compared to the sh‐Ctrl+Hb group. (B) m^6^A enrichment of *Trhr* in primary neurons showed that m^6^A peaks were decreased after Hb treatment and elevated in the context of FTO knockdown. (C) *Trhr* transcript levels in the sh‐Ctrl group, the sh‐Ctrl+Hb group, and the sh‐FTO+Hb group were quantified by RT‐qPCR. (D) Illustration of experimental process in vivo and in vitro. (E and F) Representative pictures and analysis of immunoblot showed that Trhr protein expression in the hypothalamus was elevated in the TBI groups compared to the sham group. (G) Immunofluorescence co‐staining Trhr and NeuN showed that Trhr fluorescence intensity in the hypothalamic neuron was increased in the TBI‐3d and TBI‐7d groups compared to the sham group. (H and I) Representative pictures and analysis of immunoblot showed that Trhr was increased in the sh‐Ctrl+Hb group compared to the sh‐Ctrl group, while decreased in the sh‐FTO+Hb group compared to the sh‐Ctrl+Hb group. Data are presented as mean ± SEM and were analyzed by Student's *t*‐test. ^*^ represents *p* < 0.05 and ^**^ represents *p* < 0.01. Scale bars: 25 µm.

To substantiate this speculation, we examined *Trhr* protein expression in the mouse hypothalamus after ABI in vivo and in Hb‑treated neurons in vitro (Figure 4D). Immunoblot analysis showed that TRHR protein levels were elevated in the hypothalamus following TBI compared with the sham group (Figure 4E,F). Consistent with this, immunofluorescence staining revealed increased Trhr expression in hypothalamic neurons at 3 and 7 days post‑ABI (Figure 4G). In primary neurons, Hb treatment also upregulated TRHR protein, and this effect was attenuated by FTO knockdown (Figure 4H,I), supporting the notion that TRHR expression is regulated by FTO.

To further characterize the distribution of *Trhr* in the brain, we queried the *Allen Mouse Brain Atlas* and found that *Trhr* is predominantly expressed in the LHA (Figure S6A) [20]. We also observed that *Trh*, which encodes the ligand for *Trhr*, is highly expressed in the ventral subregion of the LHA (Figure S6B). The LHA has been implicated in energy metabolism, motivated behaviors, and sleep/arousal rhythms [21, 22]. Furthermore, axonal tracing data from the Allen Mouse Brain Connectivity Atlas showed that LHA neurons project densely to the RVLM and rMR of the medulla oblongata (Figure S6C) [23]. This anatomical evidence aligns with our observation of increased c‑Fos‑positive neurons in the hypothalamus, RVLM, and rMR after ABI. Together, these data support the hypothesis that FTO‑regulated *Trhr* in the LHA may contribute to sympathetic hyperactivity following ABI.

### 2.6 *Trhr* Stability Is Regulated by M^6^A Modification

Given the established role of m^6^A in regulating RNA stability, we assessed the stability of *Trhr* mRNA across experimental groups. Transcription was inhibited with actinomycin D, and *Trhr* mRNA levels were measured at 0, 3, 6, and 9 h thereafter (Figure 5A). *Trhr* mRNA decayed more rapidly in the sh‐FTO group than in the sh‐Ctrl group (Figure 5A), indicating that FTO knockdown reduces *Trhr* transcript stability. To explore the involvement of m^6^A reader proteins in this process, we focused on the YTHDF family, key mediators of m^6^A‐dependent RNA degradation and translation. We knocked down YTHDF1, YTHDF2, or YTHDF3 using lentiviral vectors and confirmed knockdown efficiency by immunoblotting (Figure 5B,C). Subsequent experiments were conducted as outlined in Figure 5D. *Trhr* mRNA levels increased significantly only upon YTHDF2 knockdown, with minimal change in the sh‐YTHDF1 or sh‐YTHDF3 groups (Figure 5E), suggesting that FTO‐mediated regulation of *Trhr* stability depends primarily on YTHDF2. Consistent with this, TRHR protein levels were elevated in the sh‐YTHDF2 group but decreased upon YTHDF1 knockdown (Figure 5F,G), further supporting the involvement of m^6^A readers in TRHR expression. As YTHDF2 is known to facilitate mRNA decay, we hypothesized that it participates in *Trhr* degradation following FTO‐mediated m^6^A demethylation. In support of this, YTHDF2 knockdown partially rescued the destabilizing effect of FTO knockdown on *Trhr* mRNA (Figure 5H). In addition, RNA fluorescence in situ hybridization (FISH) combined with immunofluorescence revealed direct binding of YTHDF2 and YTHDF1 to *Trhr* transcripts. The number of *Trhr* RNA particles localized to neurites increased upon Hb treatment (Figure 5I,J and Figure S7A). In contrast, FTO knockdown reduced *Trhr* particle abundance in neurites and disrupted colocalization with YTHDF2 (Figure 5I,J). Together, these results demonstrate that FTO regulates *Trhr* mRNA stability in a YTHDF‐dependent manner, with YTHDF2 playing a central role.

**FIGURE 5:**
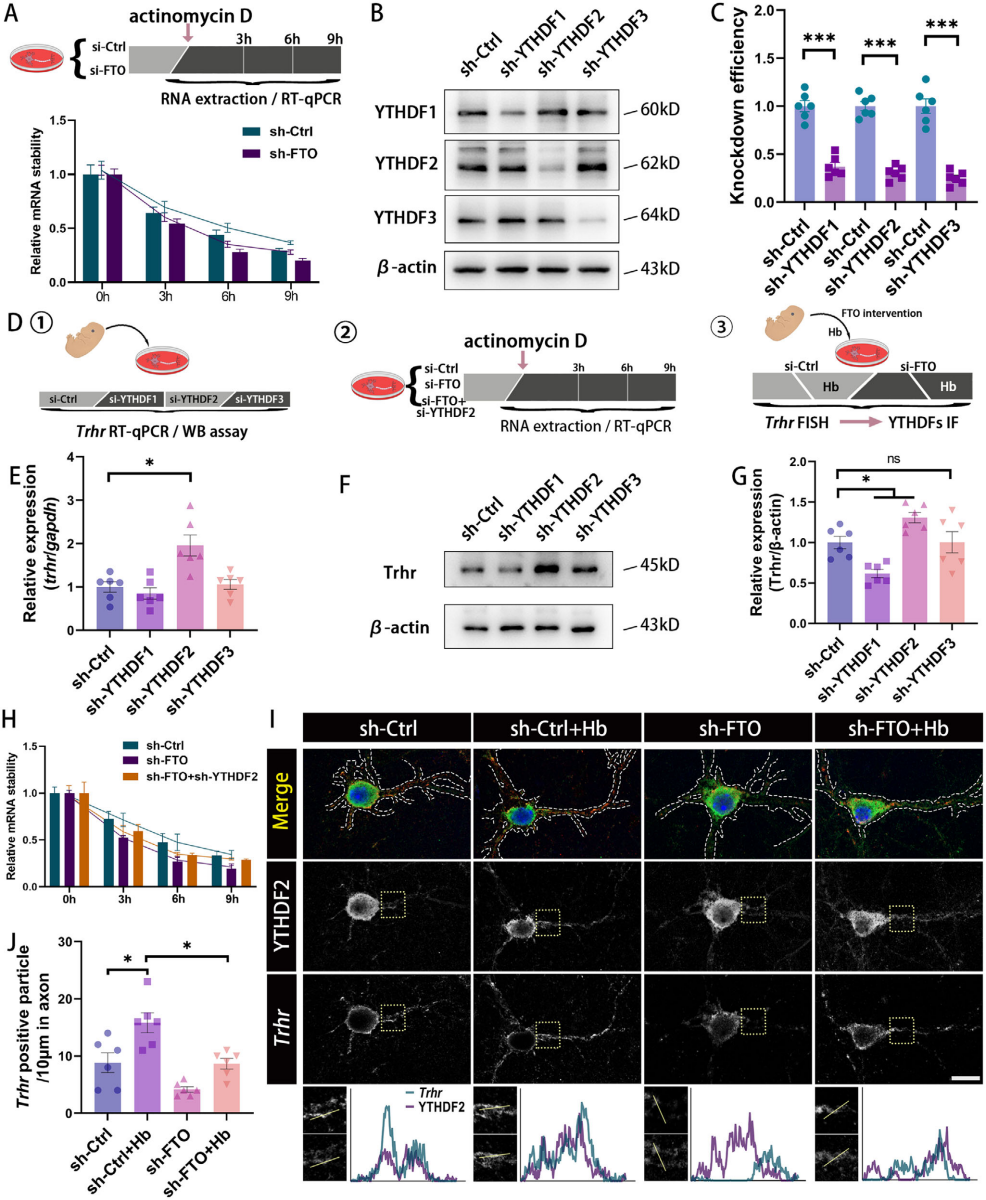
*Trhr* stability is regulated by m^6^A modification. (A) Illustration of experimental process in vitro. Primary neurons were treated with the vehicle lentivirus or lentivirus targeting FTO. After actinomycin D treatment for 3, 6, and 9 h, *Trhr* transcript levels were detected by RT‐qPCR. The results showed that *Trhr* in the sh‐FTO group exhibited a faster degradation rate than the sh‐Ctrl group. (B and C) Representative pictures and analysis of immunoblot confirmed knockdown efficiency of YTHDF1–3 in the primary neuron. (D) Illustration of experimental process in vitro. (E) After the knockdown of YTHDF1–3, contents of the *Trhr* transcript were detected by RT‐qPCR. YTHDF2 knockdown significantly increased the level of *Trhr* transcript, while minor changes were observed in the sh‐YTHDF1 and sh‐YTHDF3 groups. (F and G) Representative pictures and analysis of immunoblot showed that Trhr protein level was decreased in the sh‐YTHDF1 group while increased in the sh‐YTHDF2 group. No significant change was observed in the sh‐YTHDF3 group compared to the sh‐Ctrl group. (H) RNA stability assay was conducted among the sh‐Ctrl group, the sh‐FTO group, and the sh‐FTO+sh‐YTHDF2 group. The degradation rate in the FTO knockdown neuron was relieved by jointly knocking down YTHDF2. (I and J) Fluorescence in situ hybridization combined with YTHDF2 immunofluorescence visually confirmed the existence of *Trhr* and YTHDF2 colocalization. Meanwhile, Hb increased the *Trhr* particle in the neurite, while the knockdown of FTO decreased the level of *Trhr* in the context of Hb. *n* = 6. Data are presented as mean ± SEM and were analyzed by Student's *t*‐test. ns represents no significance. ^*^ represents *p* < 0.05 and ^***^ represents *p* < 0.001. Scale bars: 10 µm.

### 2.7 FTO‐Mediated m^6^A Modification Contributes to Sympathetic Hyperactivity After ABI

To determine whether FTO‐dependent regulation of *Trhr* influences neuronal excitability, we first measured intracellular Ca^2+^ dynamics in primary neurons. Baseline Ca^2+^ levels did not differ among the Ctrl, sh‐FTO, and sh‐FTO+sh‐YTHDF2 groups (Figure 6A). However, upon TRH stimulation, Ca^2+^ responses were attenuated in sh‐FTO neurons compared to sh‐Ctrl, and this suppression was partially rescued by concurrent YTHDF2 knockdown (Figure 6B,C). In addition, TRH‐induced upregulation of p‐ERK and c‐FOS was reduced in FTO‐knockdown neurons (Figure 6D–G). These findings indicate that FTO modulates neuronal sensitivity to TRH via m^6^A‐dependent regulation of *Trhr*.

**FIGURE 6:**
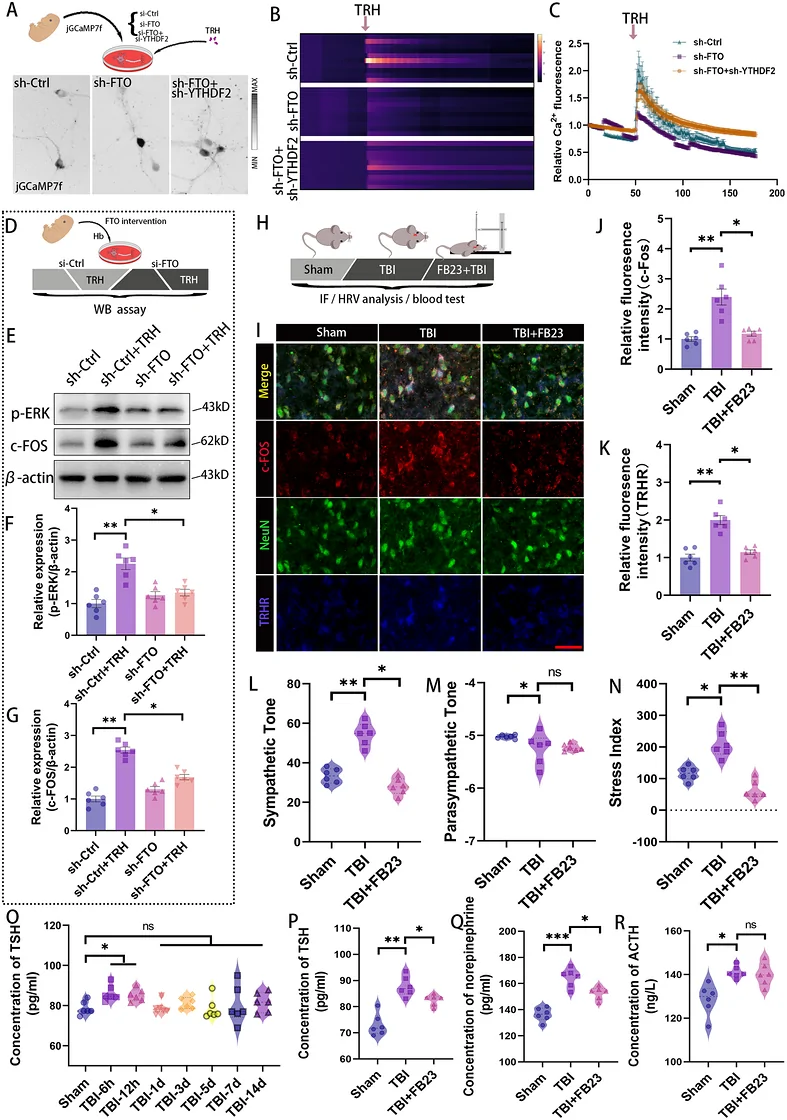
m^6^A modification by FTO participates in sympathetic hyperactivity after ABI. (A–C) Primary neurons were transfected with lentivirus encoding fast responsive Ca^2+^ fluorescent probe jGCaMP7f in advance and then allocated to the sh‐Ctrl group, the sh‐FTO group, and the sh‐FTO+sh‐YTHDF2 group. The Basal Ca^2+^ picture showed that no obvious change was observed in the sh‐Ctrl group, the sh‐FTO group, and the sh‐FTO+sh‐YTHDF2 group (A). Dynamic Ca^2+^ detection revealed neural activation among the sh‐Ctrl group, the sh‐FTO group, and the sh‐FTO+sh‐YTHDF2 group (B and C). (D) Illustration of experimental process in vitro. (E–G) Representative pictures and analysis of immunoblot showed that neuron‐activation‐related proteins c‐FOS and p‐ERK were elevated in the TRH‐treated group compared to the control group, while decreased in the sh‐FTO+TRH group compared to the sh‐Ctrl+TRH group. (H) Illustration of experimental process in vivo. (I–K) Representative images and analysis of immunofluorescence co‐staining c‐FOS, Trhr, and NeuN showed that FB23 decreased the activation of neurons and the elevation of Trhr in the hypothalamus induced by TBI. (L–N) Sympathetic tone, parasympathetic tone, and stress index were obtained by analyzing HRV from mice in the sham group, the TBI group, and the TBI+FB23 group. (O) The concentration of plasma thyroid‐stimulating hormone (TSH) from the sham group and the TBI groups was detected by ELISA. (P–R) The concentrations of plasma TSH, norepinephrine, and ACTH from the sham group, the TBI group, and the TBI+FB23 group were detected by ELISA. *n* = 6. Data are presented as mean ± SEM and were analyzed by Student's *t*‐test. ns represents no significance. ^*^ represents *p* < 0.05, ^**^ represents *p* < 0.01, and ^***^ represents *p* < 0.001. Scale bars: 25 µm.

We next evaluated the effect of FTO inhibition in vivo by administering the FTO inhibitor FB23 intracerebroventricularly prior to TBI induction (Figure 6H). Immunofluorescence analysis revealed that FB23 reduced both neuronal activation and TRHR expression in the hypothalamus post‐TBI (Figure 6I–K). Consistent with this, FB23 treatment significantly lowered sympathetic tone and stress index compared to the TBI‐only group (Figure 6L–N), suggesting that targeting FTO may alleviate ABI‐induced sympathetic hyperactivity.

To further assess *Trhr* involvement in ABI‐related pathways, we measured plasma thyroid‐stimulating hormone (TSH) levels. TSH was elevated during the acute phase but declined thereafter (Figure 6O), a pattern potentially linked to hypothalamic Trhr expression early after injury. FB23 treatment also reduced plasma TSH and NE levels in TBI mice (Figure 6P,Q). Moreover, Per1 expression—a marker of adrenal activation—was increased in the adrenal gland after TBI, and this effect was suppressed by FB23 (Figure S8A,B). Immunofluorescence further confirmed that FB23 decreased Per1^+^ signals in both adrenal and thyroid tissues (Figure S8C,D). In contrast, FB23 had minimal effect on plasma ACTH levels (Figure 6R). Together, these results demonstrate that intracranial inhibition of FTO attenuates sympathetic hyperactivity and downstream endocrine activation following ABI.

### 2.8 Lateral Hypothalamic TRHR Neurons in Peripheral Sympathetic Hyperactivity

To investigate whether TRHR neurons in the LHA modulate peripheral sympathetic outflow, we constructed AAV2/8‐*Trhr*‐Cre‐EGFP along with retrograde Cre‐dependent chemogenetic activating adeno‐associated virus (AAV2‐Retro‐hM3Dq‐Dio‐mCherry and AAV2‐Retro‐hM4Di‐Dio‐mCherry). These viruses were stereotactically co‐injected into the LHA and the RVLM, respectively, to enable cell‐type‐specific tracing and manipulation of TRHR‐expressing neurons projecting to the RVLM (Figure 7A).

**FIGURE 7:**
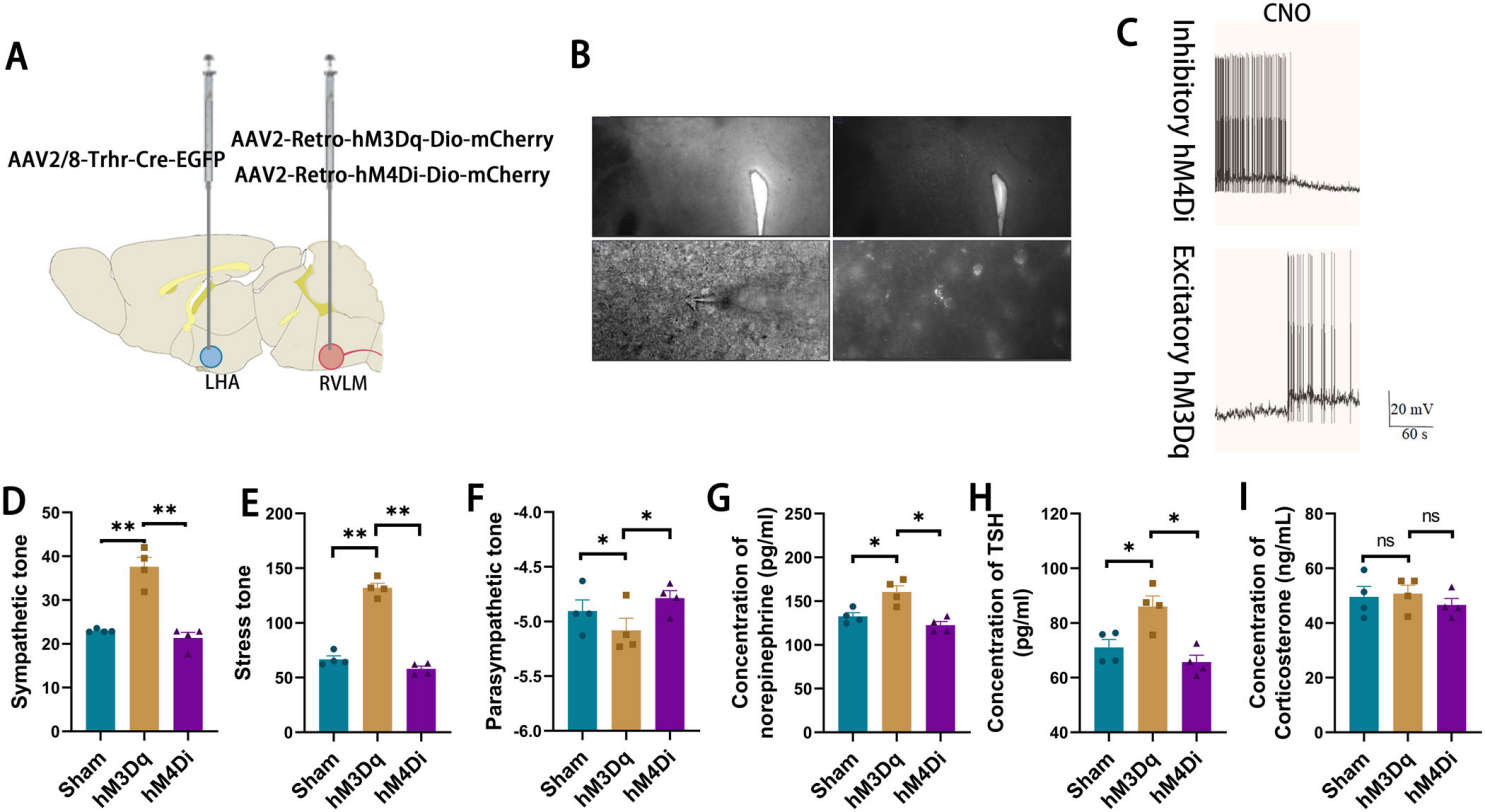
Lateral hypothalamic TRHR neurons in peripheral sympathetic hyperactivity. (A) Illustration of experimental process in vitro. (B) AAV2/8‐Trhr‐Cre‐EGFP and retrograde Cre‐dependent chemogenetic activating adeno‐associated virus (AAV2‐Retro‐hM3Dq‐Dio‐mCherry and AAV2‐Retro‐hM4Di‐Dio‐mCherry) were stereotactically co‐injected into the LHA and the RVLM, respectively. Fluorescent tract‐tracing was performed to map the projections of Trhr neurons in the LHA, demonstrating that these neurons are fluorescently labeled. (C) Whole‐cell patch‐clamp recordings demonstrated that perfusion of CNO activated or silenced neurons transduced with the excitatory (hM3Dq) or inhibitory (hM4Di) DREADD receptors, respectively, confirming the functionality of the chemogenetic tools. (D–F) Sympathetic tone, stress index, and parasympathetic tone were obtained by analyzing HRV from mice in the sham group, the hM3Dq group, and the hM4Di group. (G–I) The concentrations of plasma norepinephrine, TSH, and corticosterone from the sham group, the hM3Dq group, and the hM4Di group were detected by ELISA. *n* = 4. Data are presented as mean ± SEM and were analyzed by Student's *t*‐test. ns represents no significance. ^*^ represents *p* < 0.05 and ^**^ represents *p* < 0.01.

Fluorescence imaging in acute brain slices confirmed the presence of TRHR^+^ neuronal projections from the LHA to the RVLM (Figure 7B). Whole‐cell patch‐clamp recordings further demonstrated that perfusion of CNO reliably activated or silenced neurons transduced with the excitatory (hM3Dq) or inhibitory (hM4Di) DREADD receptors, respectively, confirming the functionality of the chemogenetic tools (Figure 7C). Chemogenetic activation of LHA TRHR neurons in vivo led to increased sympathetic outflow, as evidenced by elevated sympathetic tone and stress index in HRV analyses (Figure 7D–F) and higher serum NE levels (Figure 7G,H). However, we found that chemogenetic modulation of TRHR neurons in vivo had only a modest effect on plasma corticosterone levels in mice (Figure 7I). The precise mechanism underlying this observation remains to be elucidated in future studies. Together, these data demonstrate a role for LHA TRHR neurons in promoting sympathetic hyperactivity, suggesting these cells as a potential circuit‐level target for mitigating autonomic dysregulation after ABI.

### 2.9 Knockdown of Hypothalamic TRHR Alleviates Sympathetic Hyperactivity After ABI

To assess the functional role of TRHR in neuronal activation, we first knocked down Trhr expression in primary neurons (Figure 8A), confirming knockdown efficiency at the protein level (Figure 8B,C). Upon TRH stimulation, neurons with Trhr knockdown showed reduced levels of p‐ERK and c‐FOS compared to the sh‐Ctrl+TRH group (Figure 8D–F), indicating that TRHR is required for TRH‐induced neuronal activation.

**FIGURE 8:**
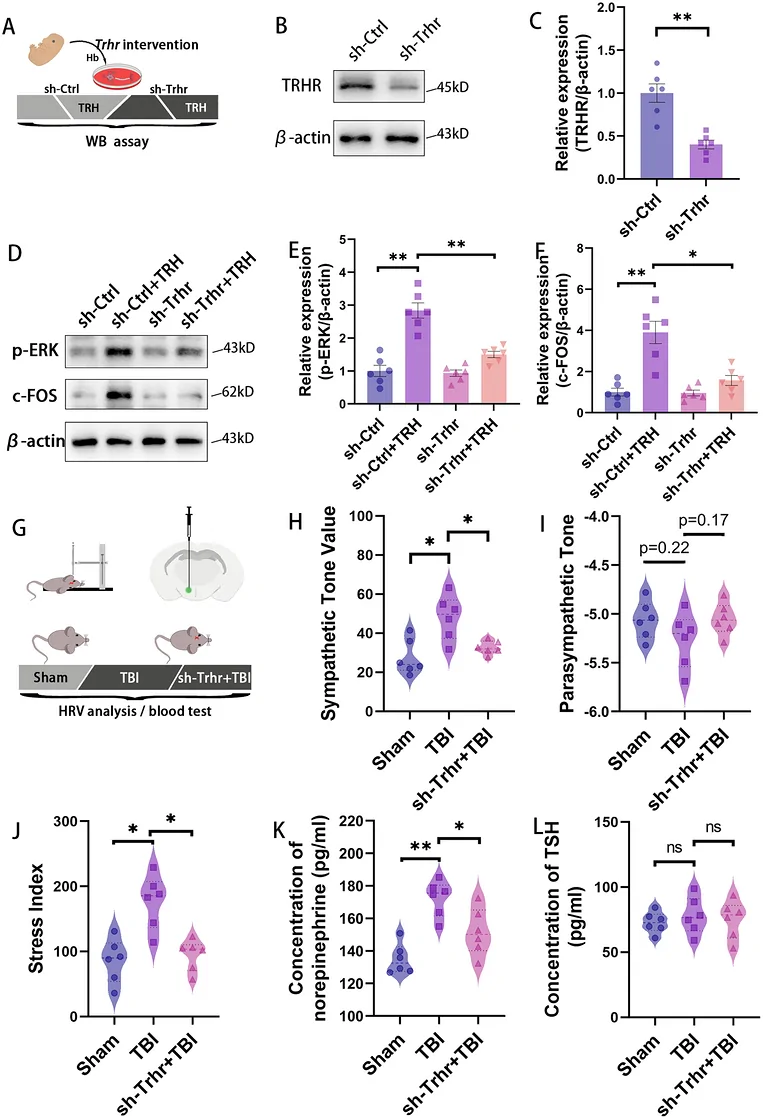
Intervention in Trhr alleviates sympathetic hyperactivity after ABI. (A) Illustration of experimental process in vitro. (B and C) Representative pictures and analysis of immunoblot assay confirmed knockdown efficiency of Trhr in the primary neuron. (D–F) Representative pictures and analysis of immunoblot assay showed that neuron‐activation‐related proteins c‐FOS and p‐ERK were elevated in the TRH‐treated group compared to the control group, while decreased in the sh‐Trhr+TRH group compared to the sh‐Ctrl+TRH group. (G) Illustration of experimental process in vivo. (H–J) Sympathetic tone, parasympathetic tone, and stress index were obtained by analyzing HRV from mice in the sham group, the TBI group, and the sh‐Trhr+TBI group. (K and L) The concentrations of plasma norepinephrine (NE) and thyroid‐stimulating hormone (TSH) from the sham group, the TBI group, and the sh‐Trhr+TBI group were detected by ELISA. *n* = 6. Data are presented as mean ± SEM and were analyzed by Student's *t*‐test. ns represents no significance. ^*^ represents *p* < 0.05 and ^**^ represents *p* < 0.01.

We next examined whether hypothalamic *Trhr* contributes to sympathetic hyperactivity in vivo. Using stereotactic delivery of AAV‐hsyn‐sh‐*Trhr* to the LHA, we knocked down *Trhr* specifically in neurons of the LHA (Figure S9A,B). HRV analysis revealed that sympathetic tone and stress index were significantly lower in sh‐Trhr+TBI mice than in TBI controls (Figure 8G–J). Plasma NE levels were also reduced following *Trhr* knockdown (Figure 8K,L), indicating that targeting hypothalamic *Trhr* ameliorates sympathetic overactivity after ABI. In summary, our findings demonstrate that FTO‐mediated m^6^A demethylation of *Trhr* in hypothalamic neurons promotes sympathetic hyperactivity after ABI (Figure S10). Therapeutic strategies aimed at modulating the FTO–*Trhr* axis may hold promise for the clinical management of autonomic dysregulation in ABI patients.

## 3 Discussion

Rapid, non‐spontaneous cascade of neurohormonal signals is the most fundamental response in human evolution to deal with damage and danger. The resulting sympathetic hyperactivity enhances physiological ability to overcome the immediate distress by influencing multiple physiological activities, including immune response, cardiovascular regulation, energy balance, etc [24]. In return, sympathetic tone also influences brain activity via cerebrovascular regulation [25], modulating the activity of astrocyte [26] and affecting glymphatic system [27]. Sympathetic hyperactivity after ABI may happen due to accommodating these compensatory needs of the central nervous system and the peripheral system. Afterward, restoration of autonomic homeostasis is attributed to intricated communication within distributed neural networks, including the hypothalamus, limbic system, and brainstem. However, prolonged and severe sympathetic hyperactivity has been widely involved in ABI patients in the ICU [28, 29], which is also observed in our patients and animal experiments, indicating inability to mitigate sympathetic surge. Eventually, long‐term sympathetic hyperactivity exerts a negative effect on the recovery of patients with ABI [30]. An increasing number of studies suggest that early use of sedatives or intervening sympathetic system may improve the prognosis of TBI and ICH patients [31, 32]. However, the difficulty in grasping the usage window for different sedatives and avoiding the potential side effects of these medicines are still concerning clinical intervention of sympathetic hyperactivity [33, 34]. Increasing evidence also indicates that modulating the autonomic nervous system plays a vital neuroprotective role following ABI. For instance, vagus nerve stimulation has demonstrated neuroprotective effects in brain injury [35]. Given the lack of molecular mechanisms underlying the occurrence of sympathetic hyperactivity, clinical management strategies have hitherto been limited.

In this study, we verified that sympathetic hyperactivity would be a common manifestation in patients and mouse model after ABI and the hypothalamic/medulla axis may be involved in sympathetic hyperactivity after ABI. Notably, we found that *Trhr* was regulated by m^6^A modification and might give rise to the occurrence of sympathetic hyperactivity after ABI. Trhr is an important hormone regulatory receptor in the hypothalamic region and plays a crucial role in regulating the function of the hypothalamus/thyroid axis which participates in the regulation of systemic blood pressure, heart rate, energy expenditure and the body's sensitivity to other hormones [36], and its activation might trigger sympathetic hyperactivity. Although the role of hormones secreted by this axis in autonomic nervous activity is gradually being revealed, the role of intracranial Trhr in autonomic nervous activity is poorly documented. An earlier literature reported that intracranial Trhr might regulating the autonomic visceral responses to acute cold via intracisternal injections of *Trhr* antisense oligodeoxynucleotides [37]. Supporting its role in autonomic activity by another indirect evidence, chlordiazepoxide, a benzodiazepine class medication, exhibiting an affinity to block Trhr [38], has the effect of treating diseases with high muscle tone or muscle stiffness, which are also symptoms of PSH [5]. Given the limited specificity of fluorescent staining for TRHR (a G‐protein‐coupled receptor), we employed a multimodal approach to better define the role of TRHR neurons within hypothalamic/medullary circuits. To this end, we combined neural tract tracing, chemogenetic manipulation, brain slice culture, and patch‐clamp recording to specifically map the projections of Trhr neurons in the LHA to the RVLM and elucidate their function in autonomic regulation. In this study, we also observed the role of hypothalamic Trhr in brain‐peripheral organs (heart, adrenal gland, and thyroid) connection and intervention with Trhr could alleviate sympathetic hyperactivity after ABI. Therefore, we speculate that *Trhr* mediated by m^6^A modification after ABI ignites the occurrence of sympathetic hyperactivity.

Mechanistically, we found that FTO participates in *Trhr* m^6^A modification after ABI, which is consistent with the fundamental function of m^6^A modification in regulation of neurological physiology and pathology [11, 12, 39]. Early research showed a close correlation between *Fto* polymorphism and obesity [40]. As research progresses, the multifunctional roles of FTO in neural function are gradually revealed. Meanwhile, the role of FTO as a demethylase has also been discovered [41]. *Fto^−/−^* mice exhibited distinctive methylation status and expression of genes in the striatum and midbrain, thereby affecting the signaling of dopaminergic neurons, leading to altered neuronal activity and behavioral responses [42]. In addition, clinical study has also shown that *Fto* polymorphism is associated with limbic system activation, which links allelic variation in *Fto* to the neural responses to caloric intake [43]. These results indicate that FTO participates in the formation and stability of neural innervation and network, which enlightens that FTO in hypothalamus may regulate Trhr‐positive neurons innervating with downstream neurons in our experiments.

In addition, posttranscriptional regulation by m^6^A modification is dependent on m^6^A‐RNA reader proteins. As the most recognized transcription and translation regulators, YTHDF proteins bind RNAs containing multiple m^6^A residues and induce phase separation of both YTHDF proteins and bound m^6^A‐containing mRNA [44]. These YTHDF paralogs have attracted increasing attention as therapeutic targets given their emerging links to human diseases, including neurological diseases [45, 46]. In this study, we found that the levels of *Trhr* mRNA and protein changed after intervention in YTHDFs, suggesting that YTHDF1–3 may regulate the stability of *Trhr* mRNA and affect its protein expression. The abilities of RNA degradation and transcriptional regulation are important mechanisms by which YTHDFs participate in the pathological processes of m^6^A‐modified mRNA. YTHDF2 was reported to have the strongest mRNA degradation ability among YTHDF1–3 [47]. Subsequent work identified a direct interaction between YTHDF2 and the CNOT1 scaffolding subunit of the CCR4‐NOT deadenylase complex, which initiated deadenylation and decay of m^6^A‐containing mRNAs [48]. In addition to recruitment of the CCR4‐NOT deadenylase complex, YTHDF2 also interacts directly with HRSP12 to recruit RNase P/MRP and elicited endonucleolytic cleavage and degradation of target mRNAs [49]. Alternatively, YTHDF2 also promotes mRNA decapping through interactions with UPF1 and recruitment of PNRC2 and DCP1A, while these interactions are not detected with YTHDF1 or YTHDF3 [50], again suggesting that YTHDF2 has a more prominent role in mRNA decay. In our study, *Trhr* mRNA stability and protein level were significantly increased during YTHDF2 knockdown, confirming that YTHDF2 regulates *Trhr* by identifying m^6^A modification. Meanwhile, after intervening with YTHDF2, our study also demonstrated that neurons enhanced responsiveness to TRH, indicating that YTHDF2 can also affect neurons' response to TRH signals.

Several limitations of this work should be acknowledged. First, while we observed increased expression of m^6^A methyltransferases alongside the demethylase FTO after ABI, the present study does not clarify how these opposing activities coordinately reshape the neuronal epitranscriptomic landscape or whether methyltransferases act independently on specific gene targets. It also remains undetermined if the elevated methyltransferases represent a compensatory response or exert other, yet uncharacterized, functions. Second, given that FTO and YTHDF2 can affect numerous mRNA species beyond *Trhr*, our data cannot exclude the possibility that their influence on sympathetic hyperactivity is partly mediated through additional signaling pathways. Third, the regulatory role of YTHDF2 in sympathetic hyperactivity was primarily investigated using in vitro approaches; direct in vivo evidence confirming its contribution is still lacking, and the precise molecular route by which YTHDF2 mediates *Trhr* mRNA decay in this context requires further elucidation. Finally, this study focused on the hypothalamic Trhr neuronal circuitry; potential contributions from other brain regions or peripheral feedback mechanisms to the observed autonomic dysregulation remain to be explored. Addressing these limitations will be essential to fully understand the mechanisms driving post‐ABI sympathetic hyperactivity and to develop targeted interventions.

## 4 Conclusion

In this study, we found that FTO‐mediated *Trhr* m^6^A modification is involved in neuronal activation, providing a possible explanation for the regulation of sympathetic hyperactivity by the hypothalamic/medullary axis after ABI. Therefore, these findings provide new insights into the epigenetic modification of hypothalamic neuron under the condition of acute damage and may enlighten medicine research to target FTO or Trhr for managing ABI‐induced automatic dysfunctions in the future.

## 5 Materials and Methods

### 5.1 Heart Rate Variability Detection and Analysis

This study was conducted in accordance with the principles of the Declaration of Helsinki. The collection of all clinical data obtained informed consent from patients or their family members and was approved by the Ethics Committee of Jinling Hospital (2023DZKY‐056‐01). ECG data were collected from patients with TBI or ICH at Jinling Hospital, with 18 healthy participants serving as the control group. The inclusion criteria were a Glasgow Coma Score (GCS) ≤ 12 and radiologically confirmed intracranial contusions or hematomas on computed tomography (CT). Patients were excluded if they had bilaterally dilated pupils upon admission or a pre‐existing history of heart disease. HRV was analyzed using Kubios software (Kuopio, Finland) to derive measures of sympathetic tone, parasympathetic tone, and the stress index [51].

### 5.2 Animals and Cells

All animal experiments were approved by the Animal Ethics Committee of Jinling Hospital (2021DZGKJDWLS‐0083). Adult C57BL/6 mice were housed under a 12‐h light/dark cycle and provided with a standard chow diet. Animals were randomly assigned to experimental groups. Anesthesia was induced and maintained with isoflurane. The TBI model in vivo was established using a weight‐drop device: A 100‐g weight was dropped from a height of 3 cm onto the exposed skull, with the impact site located 2 mm posterior to the bregma and 2 mm lateral to the midline on the right hemisphere [52]. The ICH model in vivo was induced by collagenase injection: Briefly, anesthetized mice were secured in a stereotaxic frame. After a midline scalp incision, a burr hole was drilled on the right hemisphere at coordinates 2 mm posterior to the bregma and 3 mm lateral to the midline. Collagenase was then infused at a depth of 3.5 mm below the skull surface to induce hemorrhage [53].

For stereotactic injections into the cerebral ventricle or hypothalamus, mice were anesthetized and then fastened in a stereotaxic frame. The skull was exposed through a midline incision, and a burr hole was drilled at the appropriate stereotaxic coordinates. The coordinates relative to bregma were as follows: for intraventricular injection: 0.8 mm posterior, 1.0 mm lateral; for hypothalamic injection: 0.5 mm posterior, 0.5 mm lateral. A syringe needle was then lowered to a depth of 2.5 mm (ventricle) or 4.5 mm (hypothalamus) from the skull surface. Either FB23 or an adeno‐associated virus was injected slowly. Upon completion, the needle was left in place for an additional 3 min before being slowly withdrawn to minimize reflux.

Primary neuron was extracted from fetal C57BL/6 mice (E16‐18d). Briefly, fetal mice were decapitated and rinsed with 75% ethanol. The cortex was dissected in ice‐cold Hank's balanced salt solution (HBSS) under a dissection microscope. Then, the cortex tissues were digested in HBSS containing 0.125% trypsin at 37°C for 5 min. Digestion was terminated by adding culture medium supplemented with 10% fetal bovine serum (FBS). The cell suspension was filtered through a 40 µm cell strainer and centrifuged at 1500 rpm for 5 min. The remaining was resuspended with neural culture medium (neurobasal medium with 1X B27, 1X glutamate, and 1% penicillin‐streptomycin). Cells were seeded into culture plates at an appropriate density and maintained in a 37°C incubator with 5% CO_2_. After 4 h, the medium was replaced entirely to remove non‐adherent cells. Thereafter, half of the culture medium was replaced every 3 days until the neurons reached maturity.

### 5.3 Reagents and Antibodies

Oxyhemoglobin (Hb) was purchased from Sigma‐Aldrich (Missouri, USA). The selective FTO inhibitor FB23 (HY‐137187) and the thyrotropin‐releasing hormone (TRH, HY‐P0002) were purchased from MedChemExpress Co. (New Jersey, USA). Anti‐FTO (ab280081) antibody was obtained from Abcam (Cambridge, MA, USA). Anti‐NeuN (MAB377X) antibody was purchased from EMD Millipore (Billerica, MA, USA). Anti‐p‐ERK (#4370) and anti‐β‐actin (#4970) antibodies were purchased from Cell Signaling Technology (Danvers, MA, USA). Anti‐Trhr (NBP2‐24726SS) antibody was purchased from Novus Biologicals (Littleton, CO, USA). The following antibodies were obtained from Proteintech Inc. (Rosemont, MA, USA): anti‐YTHDF1 (17479‐1‐AP), anti‐YTHDF2 (24744‐1‐AP), anti‐YTHDF3 (25537‐1‐AP), anti‐Mettl3 (15073‐1‐AP), anti‐WTAP (60188‐1‐Ig), anti‐m^6^A (68055‐1‐Ig), anti‐c‐FOS (66590‐1‐Ig), anti‐GAPDH (10494‐1‐AP), and anti‐Per1 (13463‐1‐AP).

### 5.4 Enzyme‐Linked Immunosorbent Assay (ELISA)

Serum concentrations of NE, ACTH, corticosterone, and TSH were quantified using commercial ELISA kits (Changyi Bio Co., Hunan, China). Blood plasma was collected from mice across experimental groups, and the assays were performed according to the manufacturer's instructions. Absorbance was measured with a microplate reader, and analyte concentrations were calculated based on a standard curve.

### 5.5 Cell Transfection

To knock down *Fto*, *YTHDF1*, *YTHDF2*, and *YTHDF3* expression in neurons, lentivirus or adeno‐associated virus carrying shRNA targeting respective genes and a negative control lentivirus were designed and generated by GeneChem Co. Ltd. (Shanghai, China). The primer sequence of *Fto*: 5′‐GTCTCGTTGAAATCCTTTGAT‐3′. The primer sequence of *YTHDF1*: 5′‐GGGTTGATTGTTGCATCTTTA‐3′. The primer sequence of *YTHDF2*: 5′‐GCAAACTTGCAGTTTATGTAT‐3′. The primer sequence of *YTHDF3*: 5′‐GAACCGGCTTCAACCAGAA‐3′. The primer sequence of *Trhr*: 5′‐CCCATCTACCTGCTGGACTTT‐3′. The transfection processes were conducted according to the manufacturer's instructions. The knockdown efficiency of each group was detected via immunoblot.

### 5.6 Immunoblot

Protein samples from tissues and cells were collected and quantified according to the manufacturer's instructions (Protein Extraction Kit, Beyotime Biotechnology). The immunoblot steps have been widely reported. The blots were displayed with a chemiluminescent detection kit (P90720, Millipore, MA, USA) and analyzed by ImageJ software.

### 5.7 Immunofluorescence

Cells were seeded onto coverslips and treated as indicated. Mouse brains were obtained, fixed, and embedded in paraformaldehyde for immunofluorescence staining. Tissue slides (20 µm) were generated with a freezing microtome (Leica, German). Briefly, the slides and coverslips were fixed with 4% paraformaldehyde for 15 min and then treated with 0.3% Triton X‐100 for 10 min. After the coverslip was blocked with the goat serum for 30 min, the slides were added with specific primary antibodies and secondary antibodies in turn. Finally, 4′,6‐diamidino‐2‐phenylindole (DAPI) was used to stain the nuclei. All images were captured, quantified, and analyzed by ImageJ software and GraphPad Prism 9.0 software.

### 5.8 RNA Dot Plot

The RNA samples were isolated according to the manufacturer's guidance (R0077, Beyotime Biotechnology). 2 µL drop of RNA with same concentration in each sample was loaded onto the nylon membrane. Crosslink RNA to the membrane with UV light. After washing the membrane with TBST, incubate the membrane in the blocking buffer for 1 h at room temperature. Discard the blocking buffer, and incubate the membrane with the primary antibody overnight at 4°C. Subsequently, the appropriate secondary antibody was added to incubate the membrane. After washing the membrane with TBST, incubate the membrane with SuperKine ECL substrate (Abbkine, Georgia, USA) and expose the membrane with the imaging system.

### 5.9 Methylated RNA Immunoprecipitation Sequencing

Total RNA was abstracted, purified, and quantified. The RNA integrity was assessed by Bioanalyzer 2100 (Agilent, CA, USA) with RIN number > 7.0, and confirmed by electrophoresis with denaturing agarose gel. Poly (A) RNA is purified from 30 µg total RNA using Dynabeads Oligo (dT)25‐61005 (Thermo Fisher, USA). RNA samples were then fragmented with Magnesium RNA Fragmentation Module (NEB, cat. e6150, USA) at 86°C for 7 min. An m^6^A antibody (202003, Synaptic Systems, Germany) was employed to immunoprecipitate the fragmented RNA. The Magna MeRIP m^6^A Kit (Merck Millipore) was used to enable identification and transcriptome‐wide profiling of m^6^A RNA methylation sites. High‐quality RNA‐seq libraries were prepared by using The KAPA RNA HyperPrep Kits (Roche). Sequencing was performed on an Illumina NovaSeq 6000, and sequencing reads were mapped to the mouse genome by using HISAT2 v101 software [54]. Subsequently, m^6^A peaks and m^6^A‐binding motifs were annotated, visualized, and analyzed by R package exomePeak [55], ChIPseeker [56], and edgeR [57]. Then StringTie was used to perform expression level for all mRNAs from input libraries by calculating FPKM (total exon fragments/mapped reads [millions] × exon length [kilobases]) [58]. The differentially expressed mRNAs between different groups were selected with log2 (fold change) > 1 or log2 (fold change) < −1 and *p* < 0.05.

### 5.10 RNA Stability Assay

The stability assay was conducted by adding actinomycin D (5 µg/mL) to terminate mRNA transcription in the cultured neurons. After 0, 3, 6, and 9 h of actinomycin D treatment, cells were collected for RNA isolation, and residual levels of *Trhr* were measured by RT‐qPCR. The primer information for *Trhr* is as follows: *Trhr* forward primer GTCAGTGAAATGAACCAAACCG; *Trhr* reverse primer CACAATGCCCAGTCCACAAATA.

### 5.11 Fluorescence In Situ Hybridization

FISH was performed using the RNASweAMI assay kit (Servicebio, Wuhan, China). Probe sets targeting *Trhr* were obtained from Servicebio. In brief, primary neuron cultures were fixed, washed, and subsequently permeabilized for 15 min with the provided detergent solution. Probe sets targeting *Trhr* were applied in 1:100 dilution for 3 h at 40°C followed by washes at room temperature after each step. Branched DNA reagents and fluorescence signal probe were diluted in 1:100 and incubated for 45 min in turn. FISH‐stained neurons were blocked for 30 min in goat serum at room temperature and followed with steps of fluorescence staining.

### 5.12 Lentivirus Transfection and Dynamic Monitoring of Ca^2+^ in Living Cells

Lentivirus encoding fast responsive Ca^2+^ fluorescent probe jGCaMP7f was obtained from OBiO Technology Corp. Ltd. (Shanghai, China). The transfection process proceeded according to the manufacturer's instructions. After axonal baseline Ca^2+^ fluorescence intensity was recorded for 1 min, 20 µM Ca^2+^ or 5 µM TRH was added to the culture, and constant images were recorded for 2 min with an inverted microscope (Zeiss, Germany).

### 5.13 Whole‐Cell Patch‐Clamp and Brain Slice Recording

Acute brain slices (320 µm thick) were obtained from recovered animals 3 weeks after microinjection using a Leica VT1000S vibratome. The slices were immediately transferred into ice‐cold cutting solution with the following composition (in mM): 100 glucose, 75 NaCl, 26 NaHCO_3_, 2.5 KCl, 2 MgCl_2_·6H_2_O, 1.25 NaH_2_PO_4_·6H_2_O, and 0.7 CaCl_2_. After preparation, slices were incubated for 30 min at 30°C for recovery and then transferred to recording artificial cerebrospinal fluid (aCSF) containing (in mM): 119 NaCl, 26.2 NaHCO_3_, 10 glucose, 2.4 CaCl_2_, 1.8 KCl, 1.2 MgCl_2_·6H_2_O, and 1.0 NaH_2_PO_4_·6H_2_O. The recording chamber was maintained at 30°C and continuously perfused with aCSF saturated with 95% O_2_ and 5% CO_2_.

Patch electrodes were filled with a solution containing (in mM): 140 K‐gluconate, 0.1 CaCl_2_, 2 MgCl_2_, 1 EGTA, 2 K_2_ATP, 0.1 Na_3_GTP, and 10 HEPES (pH adjusted to 7.25). Electrode resistance ranged between 4 and 6 MΩ. Voltage and current signals were recorded using an Axopatch 700B amplifier (Axon) coupled to a Digidata 1322A interface (Axon). Data were digitized and stored using pClamp software (version 9, Axon). Resting membrane potential and action potentials were measured under current‐clamp mode. Spontaneous excitatory postsynaptic currents (sEPSCs) were recorded in 10 s sweeps at a holding potential of −70 mV under the voltage‐clamp mode in the presence of 10 mM picrotoxin. Series resistance (R_s_) was monitored throughout the experiment by measuring the instantaneous current in response to a 5 mV voltage step. Although no R_s_ compensation was applied, recordings were excluded if R_s_ changed by more than 15%.

### 5.14 Statistics

All data were analyzed using GraphPad Prism 9.0 and are presented as the mean ± SEM. Statistical comparisons between two groups were performed using an unpaired Student's *t*‐test, while comparisons among multiple groups were analyzed by one‐way ANOVA followed by a post hoc test. A *p*‐value of less than 0.05 was considered statistically significant.

## Author Contributions

**Maoxing Fei**: conceptualization, methodology, data curation, formal analysis, investigation, validation, visualization, writing – original draft. **Chaochao Gao**: formal analysis, investigation. **Shiqiao Luo**: investigation, methodology. **Xiwen Huang**: investigation. **Yanling Han**: investigation. **Mengliang Zhou**: supervision. **Zhenxing Li**: conceptualization, funding acquisition, project administration, writing – review and editing. **Handong Wang**: conceptualization, funding acquisition, resources, supervision, writing – review and editing. All authors have read and approved the final manuscript.

## Ethics Statement

The acquisition of clinical data obtained consent from the patients or family members and approval from the Ethics Committee of Jinling Hospital (2023DZKY‐056‐01), in accordance with the principles of the Declaration of Helsinki. Animal experiments were approved by the Animal Ethics Committee of Jinling Hospital according to the criteria outlined in the “Guide for the Care and Use of Laboratory Animals” (2021DZGKJDWLS‐0083).

## Conflicts of Interest

The authors declare no conflicts of interest.

## Supporting information




**Supporting File 1**: mco271036‐sup‐0001‐SuppMat.pdf

## Data Availability

The raw m^6^A sequencing data have been deposited in the CNCB‑NGDC database under accession code PRJCA067702. Other data supporting the findings are included in the paper and/or Supporting Information.
