# Supplementary material for: Hypothalamus Participates in Peripheral Sympathetic Hyperactivity After Acute Brain Injury by Regulating Trhr M6A Modification
Source: MedComm (2020). 2026 Oct 5;7(10):e71036. doi: 10.1002/mco2.71036 (PMC13635787; doi:10.1002/mco2.71036)
Supplement: Supplementary file 1 [file MCO2-7-e71036-s001.pdf]

Hypothalamic *Trhr* m^{6}A modification by FTO participates in sympathetic hyperactivity after acute brain injury

Maoxing Fei[1,2], *Handong Wang*[1,3*], *Chaochao Gao*[4], *Shiqiao Luo*[3], *Xiwen Huang*[3], *Yanling Han*[1], *Mengliang Zhou*[1], *Zhenxing Li*[1*]

1. Department of Neurosurgery, Jinling Hospital, Affiliated Hospital of Medical School, Nanjing University, Nanjing, China

2. Department of Neurosurgery, Northern Jiangsu People's Hospital Affiliated to Yangzhou University, Yangzhou, China

3. Department of Neurosurgery, The Affiliated BenQ Hospital of Nanjing Medical University, Nanjing, China

4. Department of Neurosurgery, The Affiliated Jinling Hospital of Nanjing Medical University, Nanjing, China

[*]. These authors contributed equally to this work.

Corresponding authors：*Handong Wang*，*E-mail*：njhdwang@hotmail.com

Zhenxing Li，*E-mail*：lizhenxing001@126.com

Supplementary material

Table S1 Demographic and clinical characteristics of normal participates and patients with ABI.

Baseline characteristics	Normal participates (n=18)	TBI patients(n=16)	ICH patients(n=24)
Age, years	61.87 (39-74)	53.08 (19-75)	50.83 (17-84)
Sex			
Male	8/18 (44.4%)	12/16 (75.0%)	12/24 (50.0%)
Female	10/18 (55.6%)	4/16 (25.0%)	12/24 (50.0%)
Mean arterial pressure (mmHg)	94.82±1.975	86.31±3.608	94.06±1.426
Heart rate（bpm）	78.47±1.854	68.92±3.201	73.00±1.970
Body temperature（°C）	36.51±0.0573	37.41±0.2302	37.18±0.1265
Glasgow coma scale score	15	9.077±1.146	8.435±0.869
Mechanical ventilation	0/18 （0%）	6/16 （37.5%）	6/24 （25%）
Pupillary reactivity (unequal pupil size)	0/18 （0%）	5/16 （31.25%）	6/24 （25%）
Accompanied by intraventricular hemorrhage	0/18 （0%）	0/16 （0%）	9/24 （37.5%）
Blood glucose at admission, mmol/L	5.773±0.2506	7.138±0.4817	8.843±0.8487
Blood sodium at admission, mmol/L	139.9±0.7589	139.5±1.269	139.5±1.492
Blood pro-brain natriuretic peptide at admission, mmol/L	8.036±1.075	203.5±85.580	367.1±186.500

Data are n/N (%) or mean (SED). ABI, acute brain injury. TBI, traumatic brain injury. ICH, intracerebral hemorrhage.

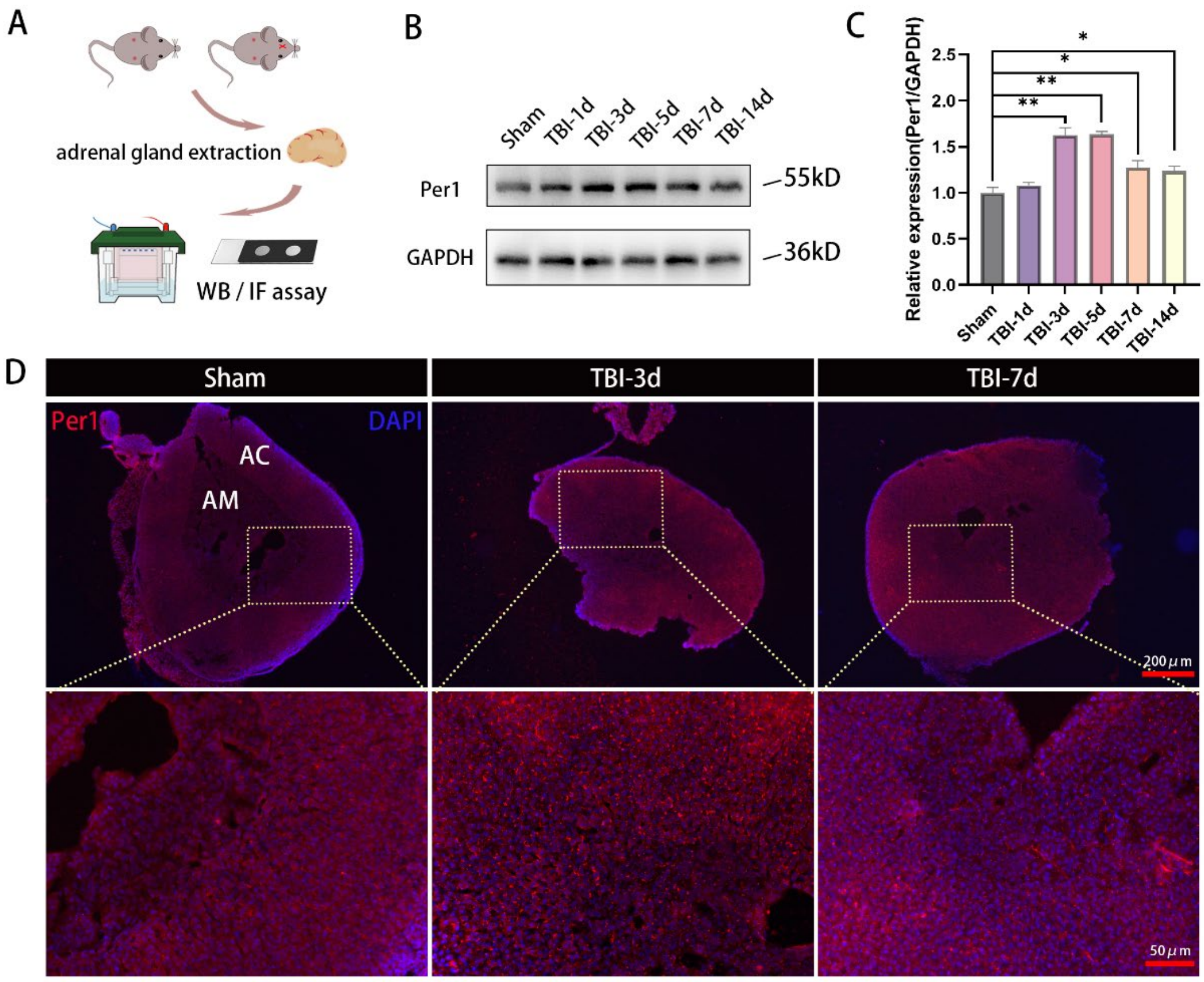

Figure S1 Activation of adrenal gland after ABI *in vivo*.

(A) Illustration of experimental process *in vivo*. **(B, C)** Representative pictures and analysis of immunoblot showed the alteration of Per1 expression in the adrenal gland after ABI *in vivo*. (**D**) Representative pictures of Perl immunofluorescence staining in mouse adrenal gland from the sham group and the TBI groups. AC, adrenal cortex. AM, adrenal medulla. n=6. Data were presented as mean ± SEM and analyzed by Student's *t* test. * represents $p<0.05$. ** represents $p<0.01$. Scale bars: 200 µm.

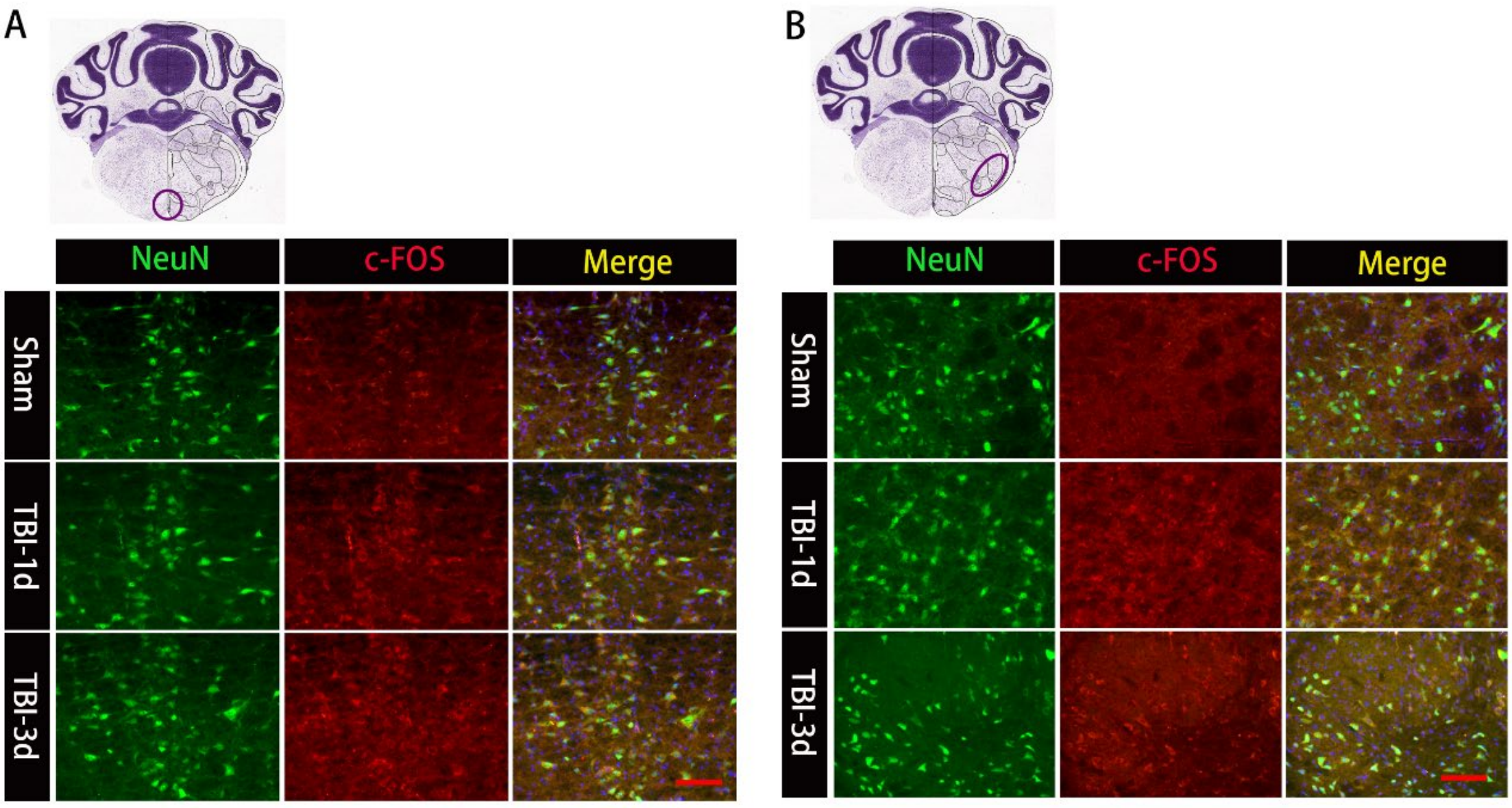

Figure S2 Neurons in the hypothalamus/ medulla oblongata axis are activated during ABI.

(A, B) The Medulla region of the sham group, TBI-1d group and TBI-3d group was stained with c-FOS. The results showed that more c-FOS positive neurons in the rostral medullary raphe region (rMR) (**A**) and rostral ventrolateral medulla (RVLM) (**B**) were observed in the TBI groups. Scale bars: 25 μm. n=6.

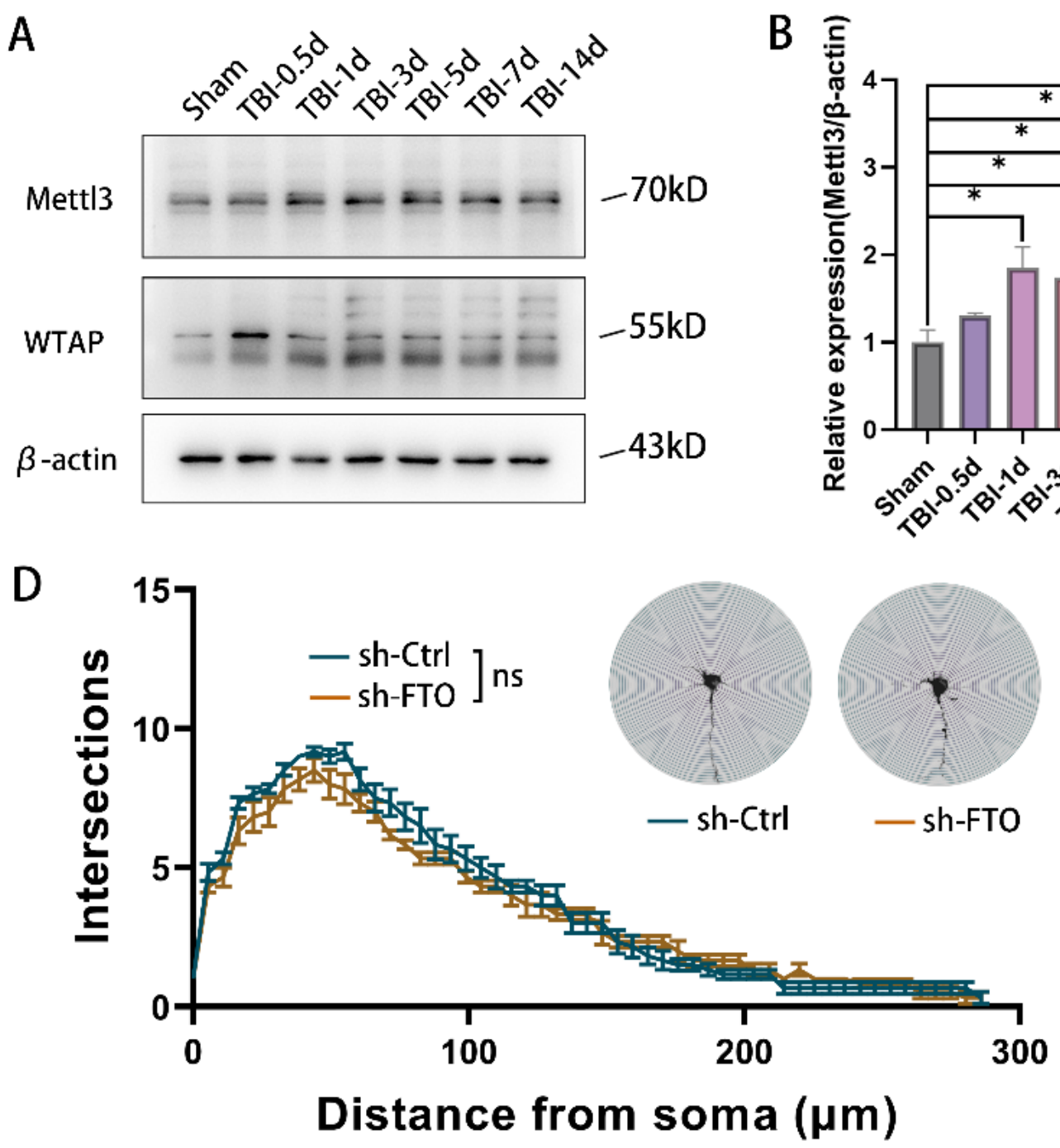

Figure S3 Alteration of m^{6}A methyltransferase in the hypothalamus after ABI *in vivo.*

(**A-C**) Representative pictures and analysis of immunoblot showed the alteration of m^{6}A methyltransferases in the hypothalamus after ABI *in vivo*. (D) Primary neurons were transfected with shRNA targeting FTO or control shRNA. Sholl analysis was performed to evaluate dendritic complexity. Knockdown of FTO did not significantly affect neuronal morphology compared with control shRNA-treated neurons. n=6. Data were presented as mean ± SEM and analyzed by Student's *t* test. ns represents no significance. * represents $p<0.05$.

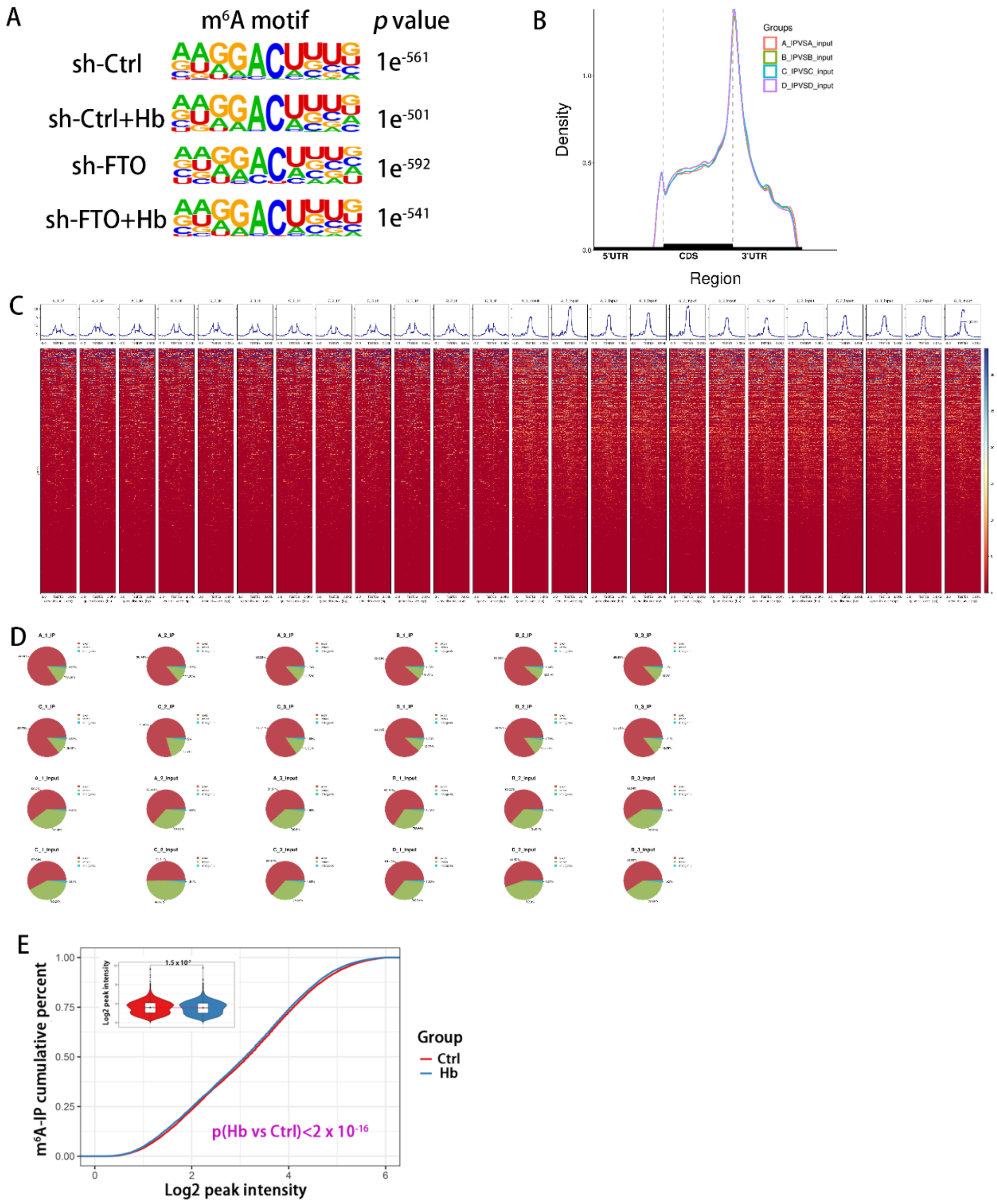

Figure S4 Changes of m^{6}A modification after oxyhemoglobin insult and FTO intervention.

(A) Consensus motif and its *p* value generated by HOMER40 of the three sets of m^{6}A sites from biological replicates of m^{6}A-CLIP-seq. **(B, C)** m^{6}A peaks were abundant in the vicinity of start and stop codons. **(D)** The m^{6}A distribution pattern was similar between the all groups. **(E)** MeRIP-seq results confirmed that m^{6}A peaks were slightly decreased in the Hb-treated group compared to the control group.

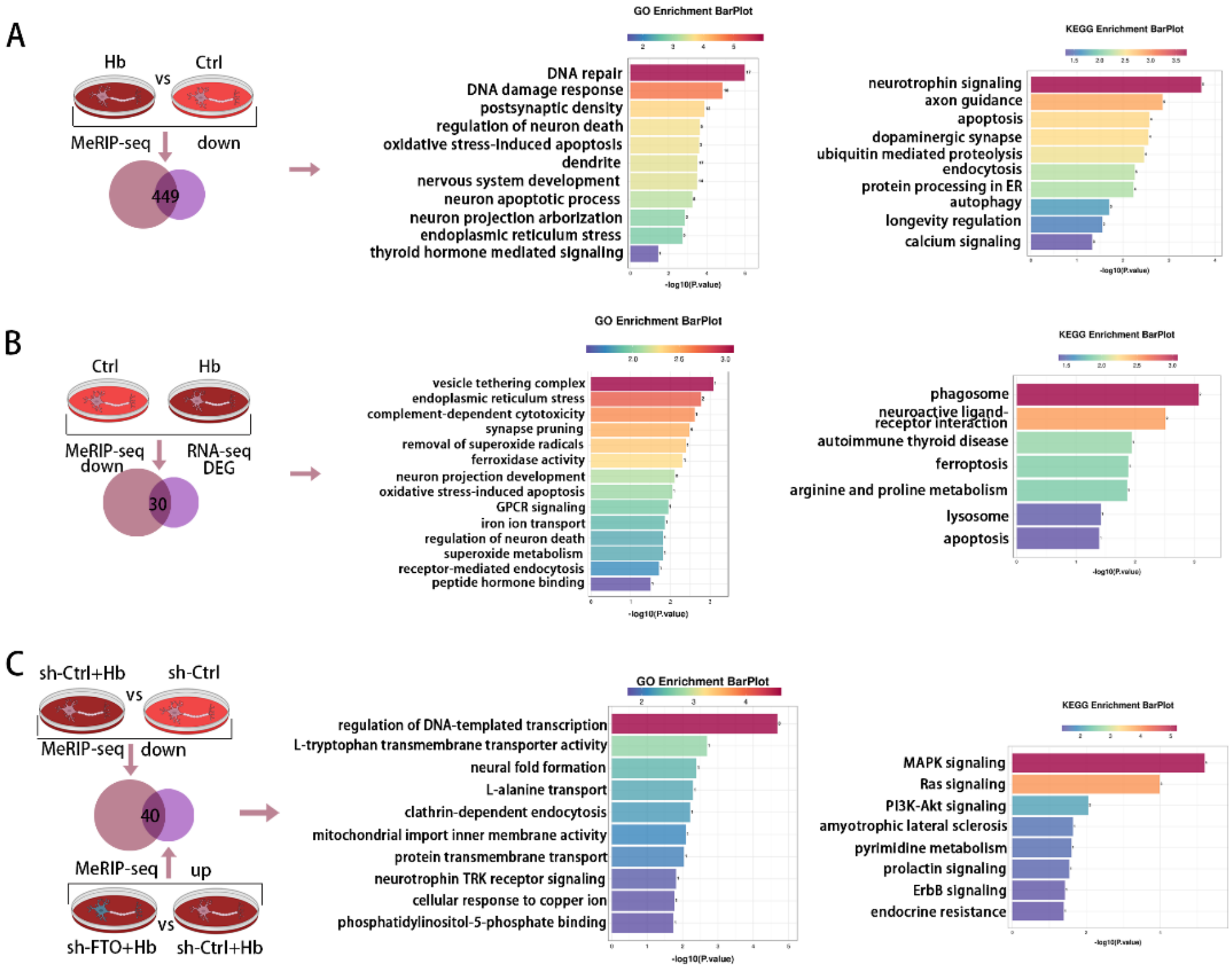

Figure S5 mRNA are massively regulated by m^{6}A after oxyhemoglobin insult. (**A-D**) Primary neurons were allocated to the sh-Ctrl group, the sh-Ctrl+Hb group, the sh-FTO group and the sh-FTO+Hb group, and then conducted MeRIP-seq. (**A**) Genome with downregulation of m^{6}A peaks in the sh-Ctrl+Hb group vs the sh-Ctrl group was identified. Then, Results of the Gene ontology (GO) pathway enrichment analysis and the Kyoto Encyclopedia of Genes and Genomes (KEGG) analysis were exhibited. (**B**) A genome with downregulation of m^{6}A peaks and RNA differential expression in the sh-Ctrl+Hb group vs the sh-Ctrl group was identified. Then, the Results of the GO pathway enrichment analysis and the KEGG analysis were exhibited. (**C**) Intersection genome between the genome (m^{6}A peaks downregulation in the Hb-treated group vs the control group) and the genome (m^{6}A peaks upregulation in the sh-FTO+Hb group vs the sh-Ctrl+Hb group) was identified. Then, the Results of the GO pathway enrichment analysis and the KEGG analysis were exhibited.

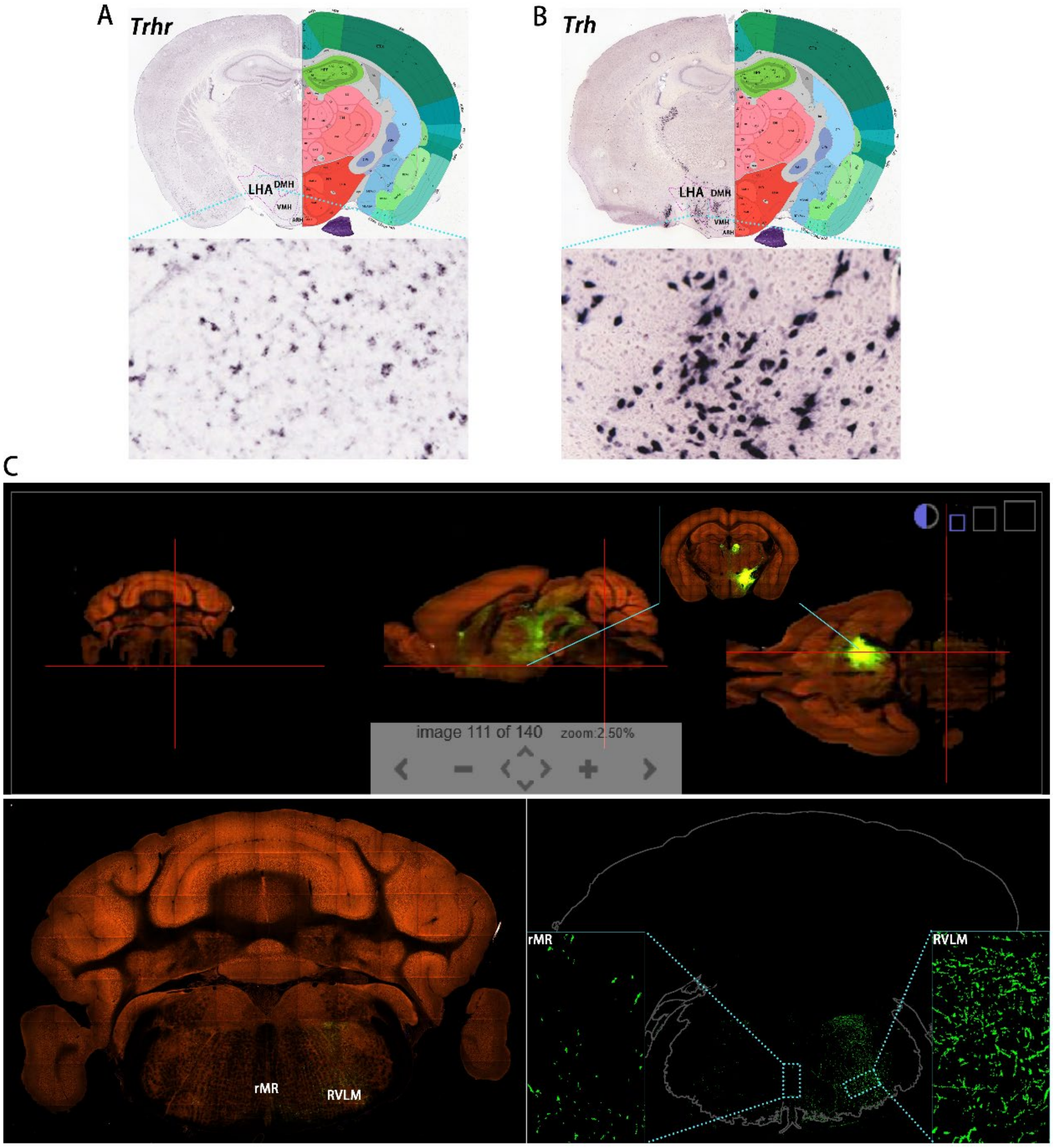

Figure S6 *Trhr* localization in the hypothalamus and related neural projection to medulla oblongata.

(A) According to the Allen Mouse Brain Atlas [1], hypothalamic *Trhr* is mainly expressed in the LHA. **(B)** According to the Allen Mouse Brain Atlas [1], *Trh* is mainly expressed in the ventral side of LHA. **(C)** According to the Allen Institute's Mouse Brain Connectivity Atlas [2], neurons in the LHA establish plenty of neural projections to the RVLM and rMR. The lateral hypothalamic area, LHA. The dorsomedial hypothalamic nucleus, DMH. VMH. The rostral ventrolateral medulla, RVLM. The rostral medullary raphe region, rMR.

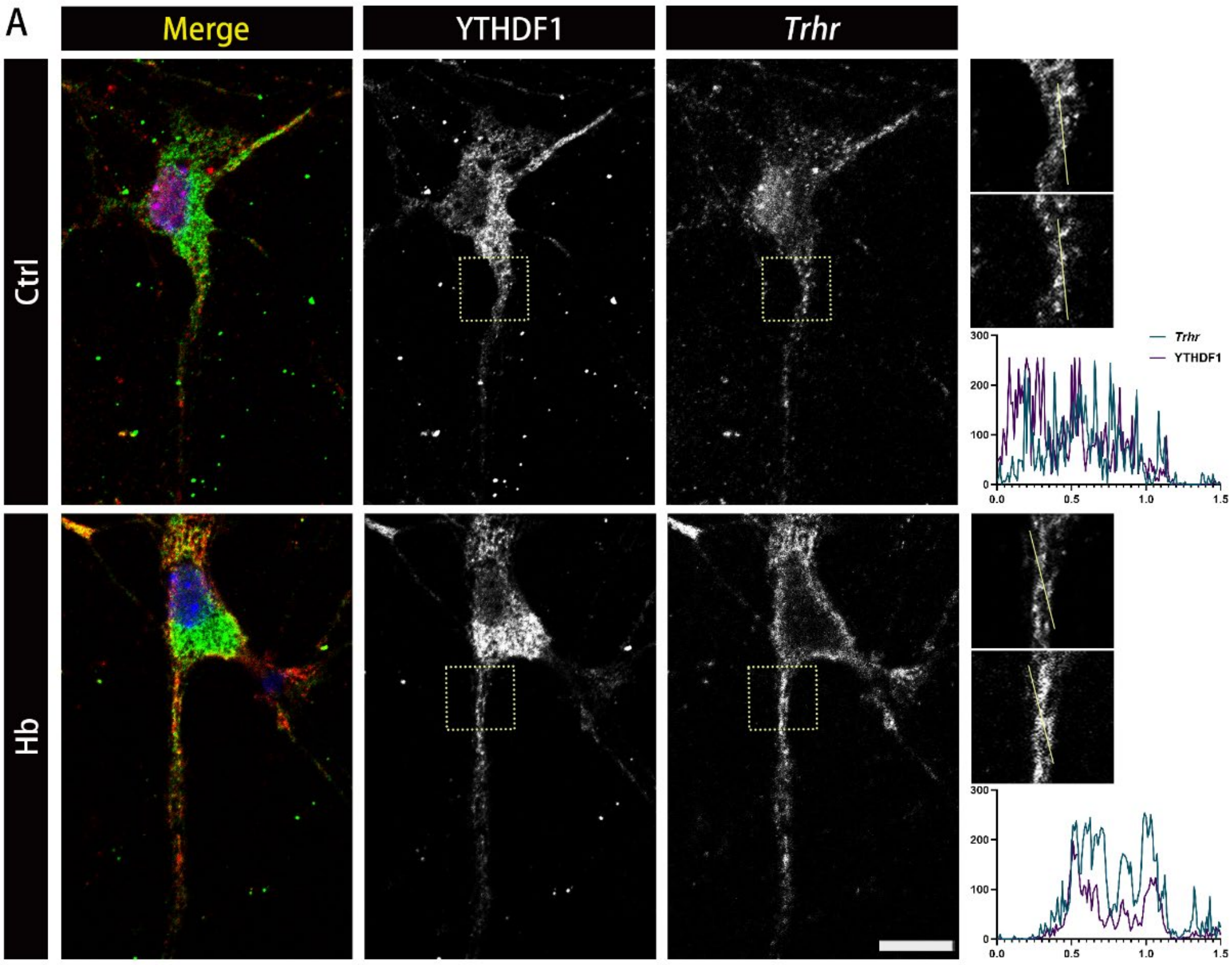

Figure S7 Colocalization of *Trhr* and YTHDF1 in the context of oxyhemoglobin insult *in vitro*.

(A) *Trhr* FISH followed by YTHDF1 immunofluorescence staining was performed. Representative pictures showed the superposition of *Trhr* and YTHDF1. Colocalization analysis was performed by image J software. Scale bars: 10 μm.

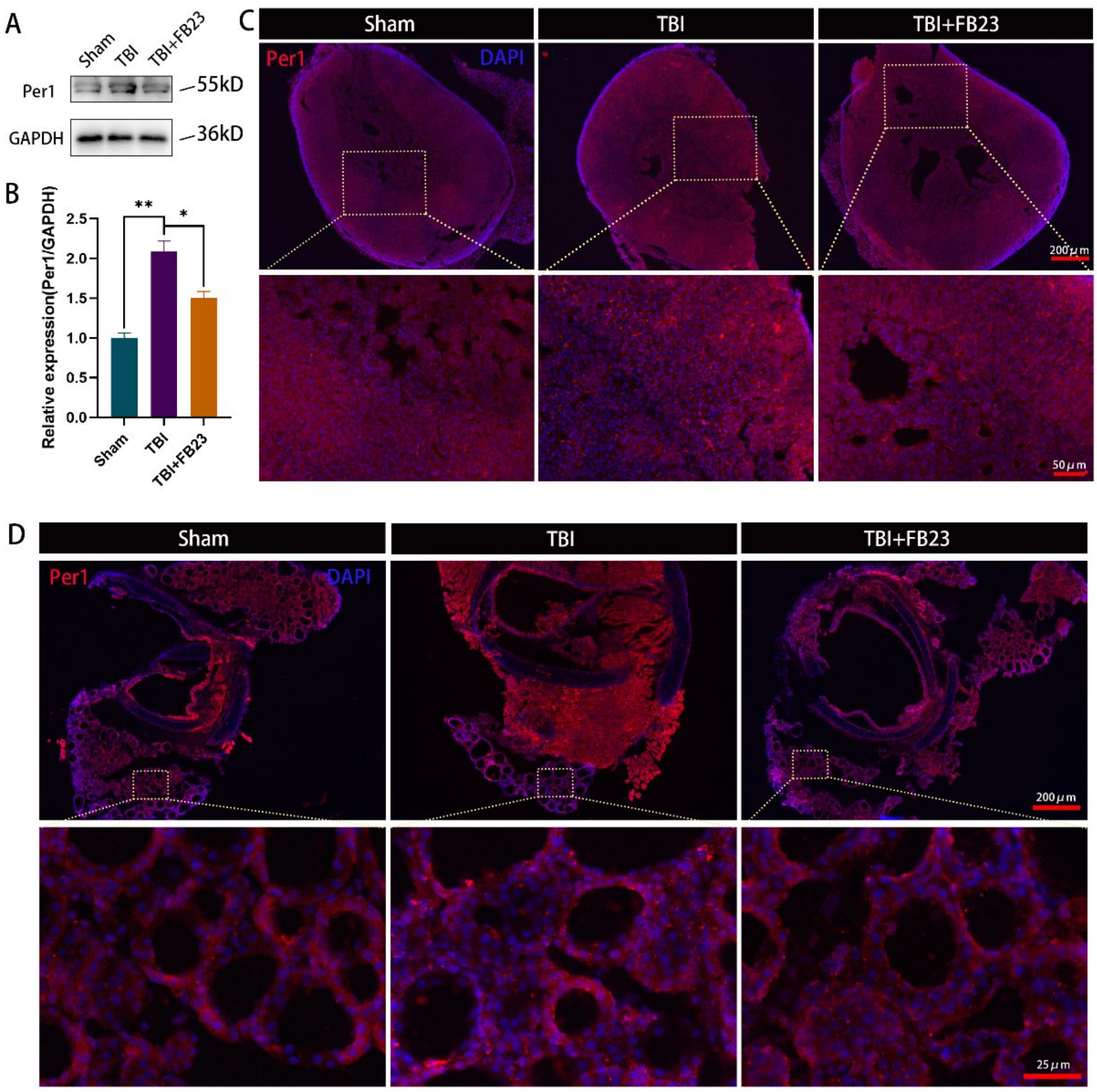

Figure S8 Activation of the adrenal gland and thyroid gland induced by ABI is alleviated by FTO inhibitor.

(A, B) Adrenal glands from the sham group, the TBI group and the TBI+FB23 group were extracted for immunoblot. Representative pictures **(A)** and analysis **(B)** of immunoblot showed the alteration of Per1 expression in the adrenal gland *in vivo*. **(C)** Representative pictures of Perl immunofluorescence staining in mouse adrenal glands from the sham group, the TBI group and the TBI+FB23 group. **(D)** Thyroid glands from the sham group, the TBI group and the TBI+FB23 group were collected for Per1 immunofluorescence. n=6. Data were presented as mean ± SEM and analyzed by Student's *t* test. * represents p<0.05. ** represents p<0.01. Scale bars: 200 μm.

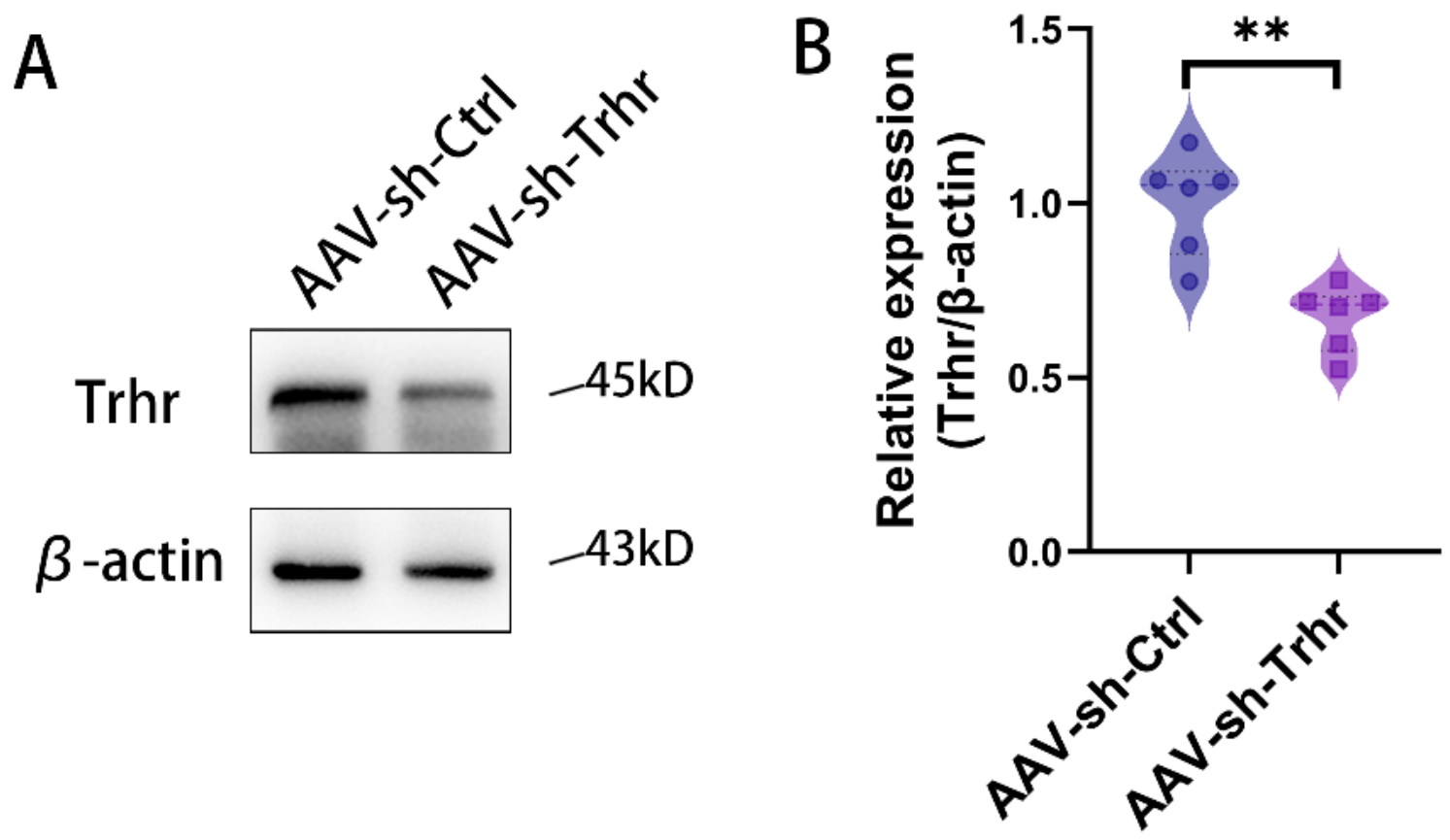

Figure S9 Verification of AAV-mediated in vivo TRHR knockdown in the LHA. Adeno-associated virus targeting *Trhr* was delivered into the LHA via stereotactic injection. Subsequent Western blot was performed on LHA tissue collected three weeks post-injection. **(A, B)** Representative pictures **(A)** and analysis **(B)** of immunoblot confirmed the knockdown efficiency of Trhr in the LHA. n=6. Data were presented as mean ± SEM and analyzed by Student's *t* test. ** represents p<0.01.

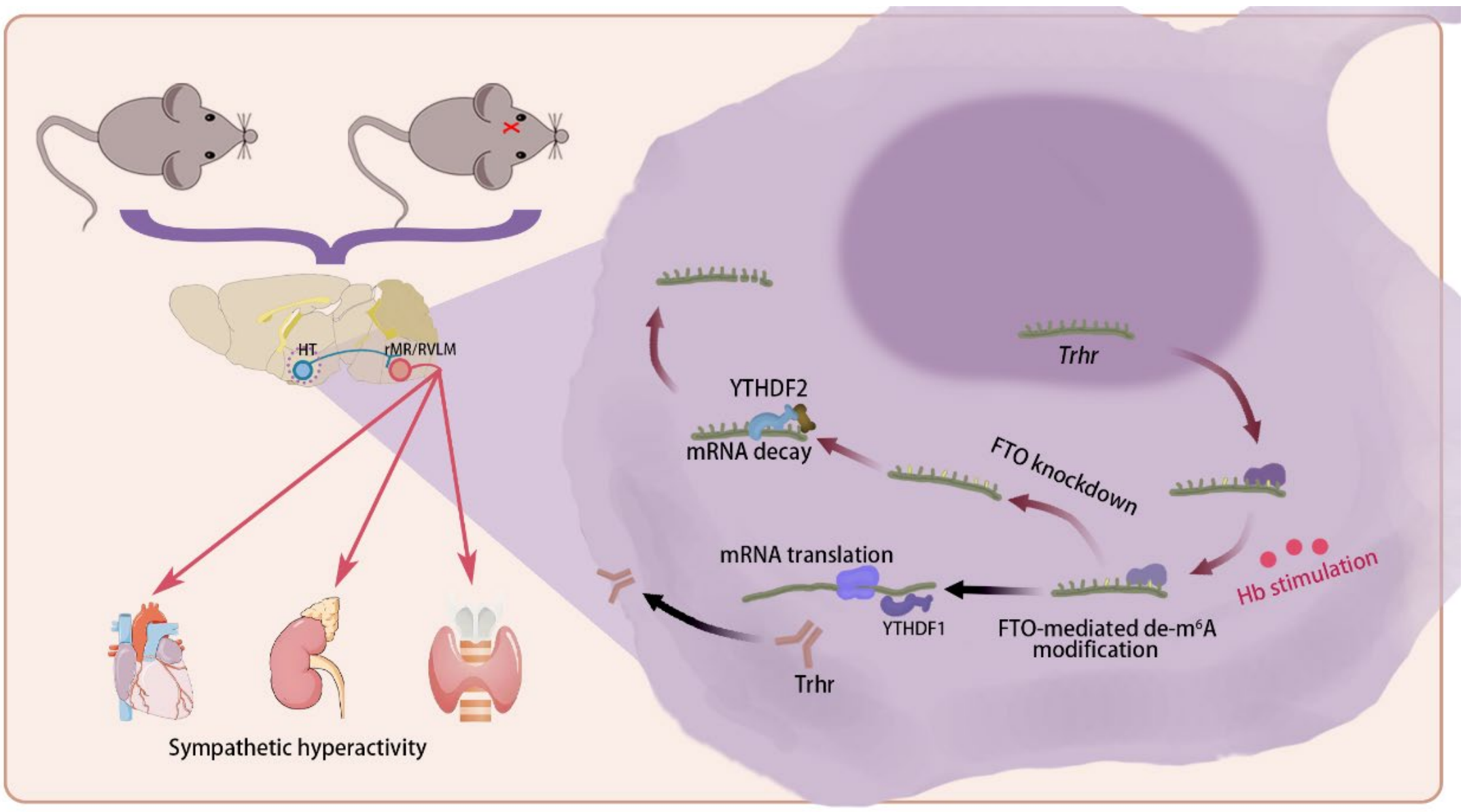

Figure S10 *Trhr* m^{6}A modification by FTO participates in sympathetic hyperactivity after acute brain injury.

Detailed flow diagram exhibiting that *Trhr* m^{6}A modification by FTO participates in sympathetic hyperactivity after acute brain injury. In this study, acute brain injury induces alteration of neural m^{6}A modification in the hypothalamus, which is involved in the occurrence of sympathetic hyperactivity through activation of the

hypothalamus/medulla axis. Mechanistically, FTO mediates m^6A modification of *Trhr* and affects its stability and translation on YTHDFs-dependent manner, thereby affecting neuronal activation. Therefore, intervening in FTO alleviates sympathetic hyperactivity induced by acute brain injury.

Reference

1. Yao Z, van Velthoven CTJ, Kunst M, et al. A high-resolution transcriptomic and spatial atlas of cell types in the whole mouse brain. *Nature*. 2023;624(7991):317-332.
2. Oh SW, Harris JA, Ng L, et al. A mesoscale connectome of the mouse brain. *Nature*. 2014;508(7495):207-14.
